# A pore-forming toxin monalysin contributes to infection-induced suppression of defecation in female *Drosophila*

**DOI:** 10.1371/journal.ppat.1014696

**Published:** 2026-10-08

**Authors:** Marko Rubinić, Yi Yu, Kathirvel Alagesan, Dagmar Frahm, Igor Iatsenko

**Affiliations:** 1 Research group Genetics of host-microbe interactions, Max Planck Institute for Infection Biology, Berlin, Germany; 2 Max Planck Unit for the Science of Pathogens, Berlin, Germany; 3 Institute of Molecular Biosciences, NAWI Graz, University of Graz, Graz, Austria; Universite Paris Descartes Faculte de Medecine, FRANCE

## Abstract

Pathogen expulsion from the gut via defecation is an important defence strategy against infection. The microbial factors that can subvert this defence reaction remain poorly understood. While many microbes have been found to increase host intestinal peristalsis, *Pseudomonas entomophila* infection in *Drosophila melanogaster* leads to infection-induced defecation blockage, particularly in females. Here, we show that this phenotype is driven by a secreted, thermosensitive protein regulated by the GacS/GacA two-component system. Proteomic comparison of the *ΔgacA* mutant, which only partially inhibits defecation, and the avirulent *Δhfq* mutant lacking RNA chaperon Hfq, which still triggers the phenotype, identified pore-forming toxin Monalysin as one of the candidate factors required for inhibiting defecation. Consistent with these findings, the Monalysin-deficient mutant failed to fully suppress defecation and exhibited attenuated virulence and reduced persistence in the gut. Hence, Monalysin besides causing intestinal damage, has a previously unknown role in suppressing defecation and pathogen expulsion. Overall, our study identified a bacterial factor that rapidly reduces defecation frequency, consistent with transient suppression of intestinal transit, thus advancing our understanding of pathogen strategies used to subvert host defences.

## Author summary

Many intestinal infections affect gut peristalsis, with increased peristalsis helping the host clear harmful microbes through defecation. However, some pathogens may benefit from slowing this process down and persisting in the intestine. We previously found that *Pseudomonas entomophila* greatly reduces defecation in female fruit flies, but the bacterial factors responsible for this effect remained unknown.

In this study, we found that *P. entomophila* releases a heat-sensitive protein that alone can reduce defecation. Using experiments with bacterial mutants and protein analysis, we identified the previously characterized toxin Monalysin as one of the key factors. Flies exposed to bacteria lacking Monalysin had more intestinal contractions, cleared bacteria more effectively, and survived infection better than those exposed to the wild-type pathogen.

Our results reveal an additional function of this bacterial toxin and show how reducing gut peristalsis can facilitate pathogen persistence in the host.

## Introduction

Intestinal peristalsis is a series of wave-like muscle contractions that move food and other gut content through the gastrointestinal tract. The primary role of intestinal peristalsis is to facilitate food digestion and nutrient absorption [1]. Waste removal and the eviction of toxins, parasites, and pathogens are additional equally important functions of intestinal peristalsis. Intestinal motility is controlled by neuronal regulation and hormonal signaling and can be affected by environmental conditions such as diet and microbial exposure [2–6].

Intestinal motility plays an important role in host-microbiota interactions and host defense against infections [7,8]. For instance, some commensal bacteria, such as *Lacticaseibacillus rhamnosus* [9] and *Lacticaseibacillus casei* [10] have been found to increase intestinal motility in mammals. Such an effect of commensal bacteria on host intestinal peristalsis is mediated either by a direct response to microbial metabolites and cell wall components, or indirectly through intestinal immune activation by microbial associated molecular patterns [8]. In mice, microbiota has been shown to provide the host with resistance against intestinal helminths via its ability to increase intestinal motility [11]. These and other studies identified a number of microbial molecules, including neurotransmitter acetylcholine and toxins driving diarrhea, that increase intestinal peristalsis [12]. However, much less is known about the microbial determinants that suppress the host intestinal peristalsis.

*Drosophila melanogaster,* thanks to its extensive genetic toolbox and manipulatable microbiota, has been used to study the molecular mechanisms of host-microbiota interactions [13–16]. Given that intestinal motility in *Drosophila* operates via principles similar to mammals, *Drosophila* provides the intestinal model system to study the general mechanisms underlying the effect of microbes on the host intestinal peristalsis [17], [18]. Several studies explored the effect of certain pathogens and commensals on *Drosophila* gut motility. For example, the Gram-negative bacterium *Pectobacterium carotovorum* –*Ecc15* secretes uracil in the gut [19]. The presence of uracil triggers a host immune reaction leading to the DUOX-dependent production of reactive oxygen species (hypochlorous acid, HOCl) which is released in the lumen [19]. Du and colleagues have shown that the evolutionarily conserved Transient Receptor Potential A1 channel (TRPA1) binds HOCl and triggers increased defecation [18]. Benguettat et al [20] extended these findings and proposed that the binding of HOCl to TRPA1 induces a calcium flux in anterior Diuretic Hormone 31 (DH31)-positive enteroendocrine cells, thus promoting DH31 release. DH31 in turn binds to its receptor, DH31-R, in the neighboring visceral muscle and triggers contractions. Such visceral muscle contractions help the host to evict opportunistic bacteria from the gut via defecation, demonstrating that pathogen expulsion via defecation is a defense mechanism against intestinal pathogens [18,20]. The same mechanism likely explains the increased defecation rate of flies infected with *Pseudomonas aeruginosa* or colonized with specific microbiota members, like *Enterococcus haemoperoxidus* [17]. Another *Drosophila* commensal *Lactiplantibacillus plantarum* was shown to promote gut motility in larvae via production of acetylcholine – a neurotransmitter with a known role in enabling muscle movement [21]. Whereas increased peristalsis upon pathogen exposure may benefit the flies by clearing the pathogens, the relevance of microbiota-mediated enhancement of peristalsis for the host physiology remains to be determined. While several bacteria have been reported to increase intestinal peristalsis and defecation in flies, whether and how pathogens suppress this defense reaction has not been elucidated.

Recently, we showed that *Pseudomonas entomophila*, a natural pathogen of *Drosophila* [22], inhibits defecation and prevents pathogen clearance from intestine in female but not in male flies [23]. Given that the enforcement of defecation frequency with chemical or genetic tools protects flies from infection [23] indicates that such blockage of defecation is an essential previously unknown aspect of *P. entomophila* pathogenesis. Several of *P. entomophila* virulence factors have been identified, including the pore-forming toxin Monalysin and secreted protease, AprA, which allows *P. entomophila* to counteract the intestinal immune response [24,25*]. P. entomophila* virulence is controlled by two global regulatory systems: the GacS/GacA two component system [22,26], and a second system called Pseudomonas virulence factor (*pvf*) – a gene cluster which encodes a secreted secondary metabolite [27,28]. However, *P. entomophila* factors inhibiting defecation and pathogen eviction remain unknown. Identification of these factors will advance our understanding of pathogen strategies used to subvert host defences.

In the present study, we used biochemical, genetic, and proteomic approaches to identify pathogen factors contributing to infection-induced suppression of defecation and pathogen clearance. We showed that the responsible component is a secreted, thermosensitive protein regulated by the GacS/GacA two component system. We employed proteomic approach to compare proteins produced by different *P. entomophila* strains that differently alter defecation in female flies. Finally, we identified Monalysin as one of the *P. entomophila* virulence factors required to suppress pathogen eviction via defecation.

## Results

### A secreted, proteinaceous factor from *P. entomophila* is sufficient to reduce defecation

To identify *P. entomophila* factor(s) causing defecation blockage, we utilized a defecation assay previously used by us and others [18,23]. All experiments were performed at 29°C, which is close to the optimal growth temperature for *P. entomophila*. We fed flies with bacteria or culture supernatant mixed with a blue color dye that allowed us to estimate the defecation frequency by scoring the number of blue-colored defecation spots deposited within the scoring period of 2 h (Fig 1A). Using this assay, we confirmed that *P. entomophila* suppresses defecation, while *Ecc15* expectedly induced it compared to control sucrose-fed flies (Fig 1B, Fig 1B’), when we compared the absolute number of defecation spots per fly (dots/fly) (Fig 1B). To account for day-to-day variability and facilitate comparison of relative changes between experimental conditions, we also expressed defecation as fold change relative to the mean defecation frequency of the sucrose control from the same experimental day (Fig 1B’). This complementary analysis confirmed inhibition of defecation by *P. entomophila* and induction by *Ecc15* (Fig 1B’).

**Fig 1:**
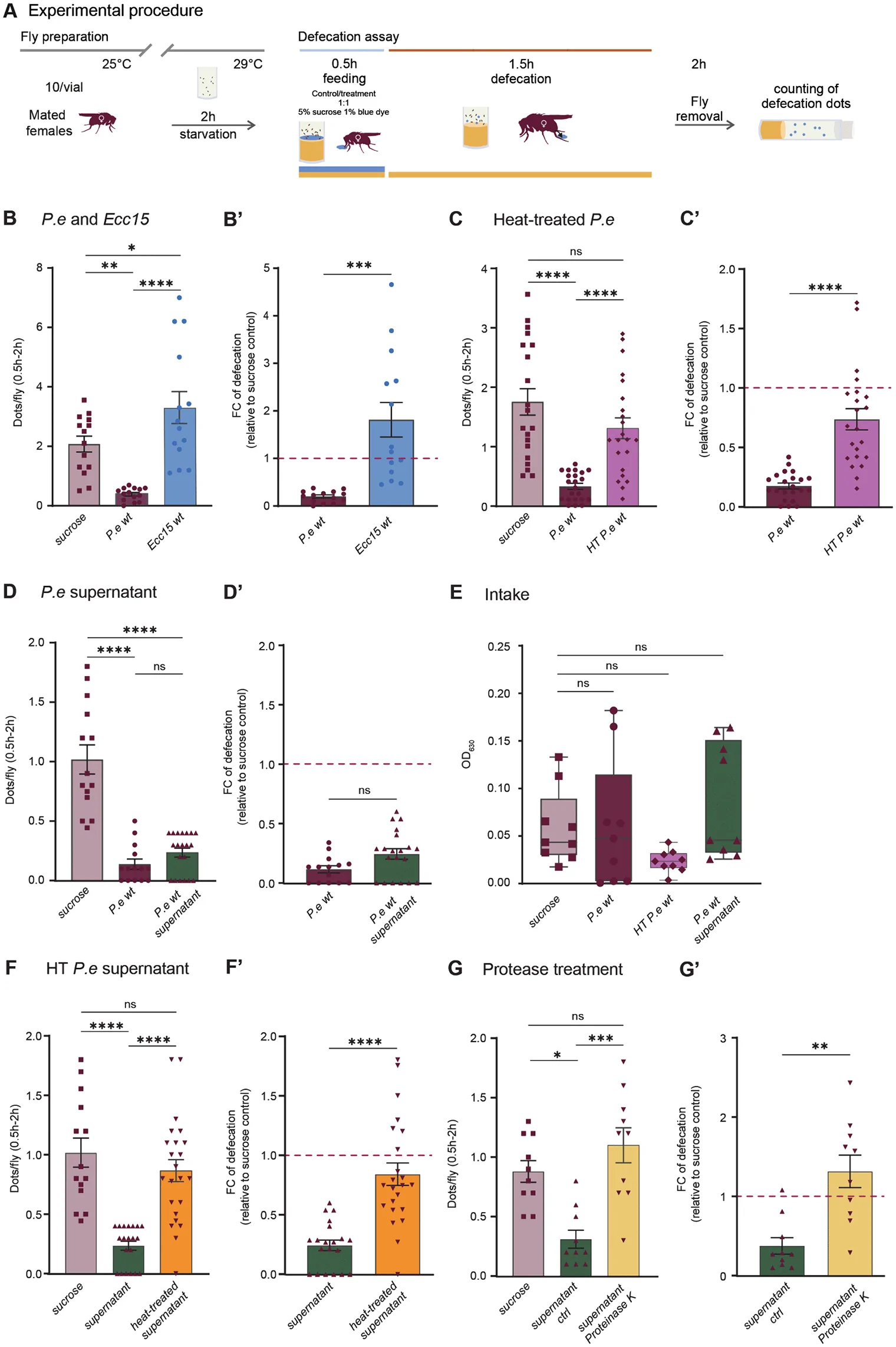
A secreted P. entomophila protein is sufficient to reduce defecation. **(A)** Graphical illustration of defecation assay. **(B and B’)** The defecation rate of female *w1118* iso flies measured 0.5 to 2 h after exposure to blue-dyed sucrose (control), *P. entomophila*, or *Ecc15* in the 0.5 h protocol. **(B)** Significance by one-way ANOVA with Šídák’s multiple comparisons test (n (sucrose) = 14, n (*P.e* wt) = 14, n (*Ecc15*) = 14, N (days) = 4). **(B’)** Fold change calculated from mean values of the sucrose control of the appropriate treatment from the same biological repeat. Significance by Welch’s unpaired t-test. N (days) indicates the number of independent experimental days on which the experiment was repeated. **(C and C’)** The defecation rate of female *w1118* iso flies measured 0.5 to 2 h after exposure to blue-dyed PBS (sucrose control), *P. entomophila* or heat-inactivated *P. entomophila* (HT) in 0.5 h protocol. **(C)** Significance by Kruskal–Wallis test with Dunn’s multiple comparisons test. (n (sucrose) = 20, n (*P.e* wt) = 22, n (HT *P.e* wt) = 22, N (days) = 4). **(C’)** Fold change calculated from mean values of PBS control of appropriate treatment from the same biological repeat. Significance by Welch’s unpaired t-test. **(D and D’)** The defecation rate of female *w1118* iso flies measured 0.5 to 2 h after exposure to blue-dyed LB (sucrose control), *P. entomophila* or *P. entomophila* cell-free culture supernatant in 0.5 h protocol. **(D)** Significance by Kruskal–Wallis test with Dunn’s multiple comparisons test. (n (sucrose) = 14, n (*P.e* wt) = 14, n (*P.e* wt supernatant) = 20, N (days) = 4). **(D’)** Fold change calculated from mean values of LB control of appropriate treatment from the same biological repeat. Significance by Mann-Whitney test. **(E)** Intake of blue-dyed sucrose (control), *P. entomophila*, heat-inactivated *P. entomophila*, or *P. entomophila* cell-free culture supernatant by female *w1118 iso* flies during 0.5 h exposure. Intake was quantified by measuring the OD_600_ of blue dye in flies following exposure. Significance by Kruskal–Wallis test with Dunn’s multiple comparisons test (n = 9 per treatment, N (days) = 3). All indicated pairwise comparisons with the sucrose control were not significant. **(F and F’)** The defecation rate of female *w1118* iso flies measured 0.5 to 2 h after exposure to blue-dyed LB (sucrose control), *P. entomophila* cell-free culture supernatant, or heat- treated *P. entomophila* cell-free culture supernatant in 0.5 h protocol. **(F)** Significance by Kruskal–Wallis test with Dunn’s multiple comparisons test. (n (sucrose) = 14, n (supernatant) = 20, n (heat-treated supernatant) = 23, N (days) = 4). **(F’)** Fold change calculated from mean values of LB control of appropriate treatment from the same biological repeat. Significance by Mann-Whitney test. **(G and G’)** The defecation rate of female *w1118* iso flies measured 0.5 to 2 h after exposure to blue-dyed LB (sucrose control), *P. entomophila* cell-free culture supernatant, or *P. entomophila* cell-free culture supernatant treated with Proteinase K in 0.5 h protocol. **(G)** Significance by Kruskal–Wallis test with Dunn’s multiple comparisons test. (n (sucrose) = 10, n (supernatant ctrl) = 10, n (supernatant Proteinase K) = 10, N (days) = 3). **(G’)** Fold change calculated from mean values of LB control of appropriate treatment from the same biological repeat. Significance by Welch’s unpaired t-test.

We tested whether *P. entomophila* must be alive to block defecation. As shown in Fig 1C, defecation frequency of flies fed with heat-inactivated *P. entomophila* was significantly higher as compared to flies fed with alive pathogen (Fig 1C, Fig 1C’). These results suggest that *P. entomophila* must be alive to cause defecation blockage. Next, we investigated the effect of *P. entomophila* cell-free culture supernatant on defecation to test whether a secreted factor is sufficient to reduce defecation. Indeed, we found that flies fed with cell-free supernatant exhibited the same degree of reduction in defecation as flies fed with the pathogen (Fig 1D, Fig 1D’). Importantly, flies ingested similar amounts of tested solutions, suggesting that defecation differences are not due to differences in intake (Fig 1E). Heat treatment of the cell-free supernatant abolished its ability to inhibit defecation (Fig 1F, Fig 1F’). Finally, incubation with Proteinase K had a similar effect (Fig 1G, Fig 1G’), supporting a proteinaceous effector. Together, these results identify the active component as a secreted thermosensitive proteinaceous factor that triggers infection-induced defecation blockage.

### The GacS/GacA system controls secretion of some of the defecation-blocking factors

We next examined bacterial regulatory systems that could control the secretion of the active peptide/protein. We started with the RNA chaperone Hfq which *plays important regulatory role in bacteria* [29]. Given that we previously showed that Hfq responds to the gut environment and is required for *P. entomophila* virulence [23], we tested whether Hfq is necessary for inhibition of defecation. As shown in Fig 2A and 2A’, infection with *Δhfq* mutant inhibited defecation to the same degree as infection with wild-type *P. entomophila.* Hence, while Hfq is necessary for virulence, it is not required for defecation blockage. Next, we tested GacS/GasA system known to control secondary metabolite production, protein secretion, and virulence [26]. The *ΔgacA* mutant still suppressed the defecation, although not as strongly as wild-type *P. entomophila* (Fig 2B, Fig 2B’). These results indicate that at least some of the factors required for infection-induced defecation blockage in female *Drosophila* are controlled by the GacS/GacA regulatory system.

**Fig 2:**
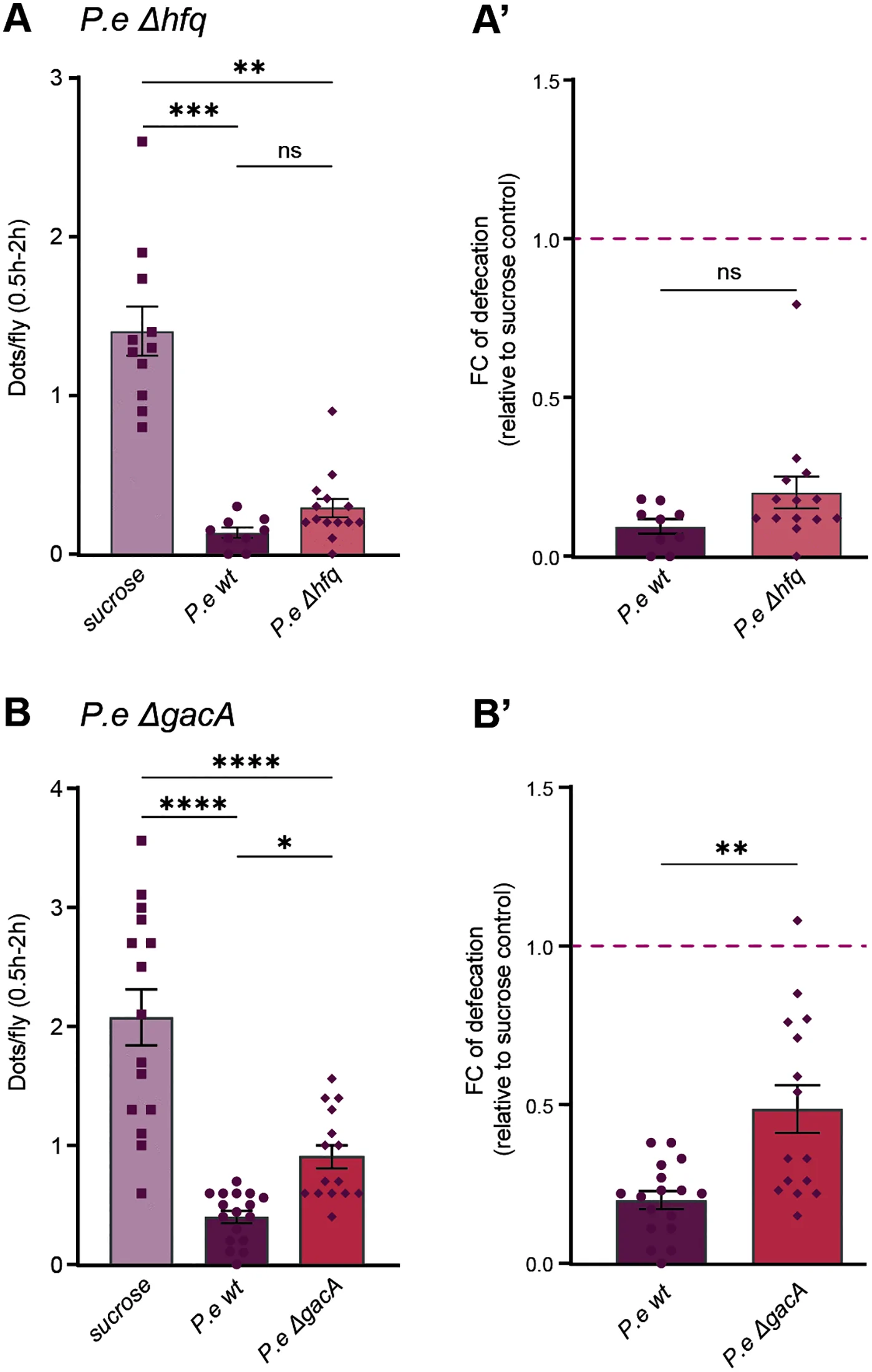
The GacS/GacA system controls secretion of the defecation-blocking factor. **(A and A’)** The defecation rate of female *w1118* iso flies measured 0.5 to 2 h after exposure to blue-dyed PBS (control), *P. entomophila* wt, or *P. entomophila Δhfq* in 0.5 h protocol. **(A)** Significance by Kruskal–Wallis test with Dunn’s multiple comparisons test. (n (sucrose) = 11, n (*P.e* wt) = 9, n (*P.e Δhfq*) = 14, N (days) = 3). **(A’)** Fold change calculated from mean values of PBS control of appropriate treatment from the same biological repeat. Significance by Mann-Whitney test. **(B and B’)** The defecation rate of female *w1118* iso flies measured 0.5 to 2 h after exposure to blue-dyed PBS (control), *P. entomophila* wt, or *P. entomophila ΔgacA* in 0.5 h protocol. **(B)** Significance by one-way ANOVA with Šídák’s multiple comparisons test. (n (sucrose) = 15, n (*P.e* wt) = 17, n (*P.e ΔgacA*) = 15, N (days) = 4). **(B’)** Fold change calculated from mean values of PBS control of appropriate treatment from the same biological repeat. Significance by Welch’s unpaired t-test.

### Comparative proteomics identified Hfq- and GacA-regulated proteins

To proceed further with the identification of protein(s) causing defecation suppression, we compared proteomes of *P. entomophila* wild-type, *ΔgacA,* and *Δhfq* mutants grown in LB medium. Comparing these three strains allows us 1) to identify GacA- and Hfq-controlled proteins, 2) decouple virulence and defecation-inhibiting factors, as while both *ΔgacA* and *Δhfq* mutants are avirulent, only *ΔgacA* is impaired in the ability to inhibit defecation.

Using a log2 fold-change cutoff of 1.5, we identified 531 proteins with increased and 108 with decreased abundance in the *Δhfq* mutant relative to wild-type *P. entomophila* (Fig 3A, S1 Table). We noticed several virulence factors, such as protease AprA, an esterase EstA, flagellin FliC, *Pseudomonas virulence factor* PvfA, PvfB, PvfC among proteins with increased abundance in *Δhfq* mutant. To obtain an overview of the functionality of the proteins up-regulated in *Δhfq* mutant, we performed gene ontology (GO) annotation enrichment analysis. The following GO terms were identified (S1A Fig): Localization and transport (incl. transmembrane and ABC transporter activity), organic acid and small molecule metabolism, oxidoreductase activity, catabolic processes (organic substance, organonitrogen compound, carboxylic acid), fatty acid metabolism and secondary metabolite biosynthesis. Hence, loss of Hfq increases abundance of proteins linked to transport and metabolic turnover. Several virulence factors, such as catalase KatA [30], pilin pilH [31], isocitrate lyase AceA [32], alginate biosynthesis proteins AlgA, AlgD, AlgE, AlgZ [33] were detected among proteins down-regulated in *Δhfq* mutant (Fig 3A, S1 Table). The following GO terms were enriched among the down-regulated proteins (S1B Fig): cell envelope organization, oxidoreductase activity acting on metal ions, response to stress, starch and sucrose metabolism, ferroxidase activity. Thus, reduced abundance of proteins involved in stress response and redox balance, suggests that Hfq contributes to oxidative and cell envelope stress response.

**Fig 3:**
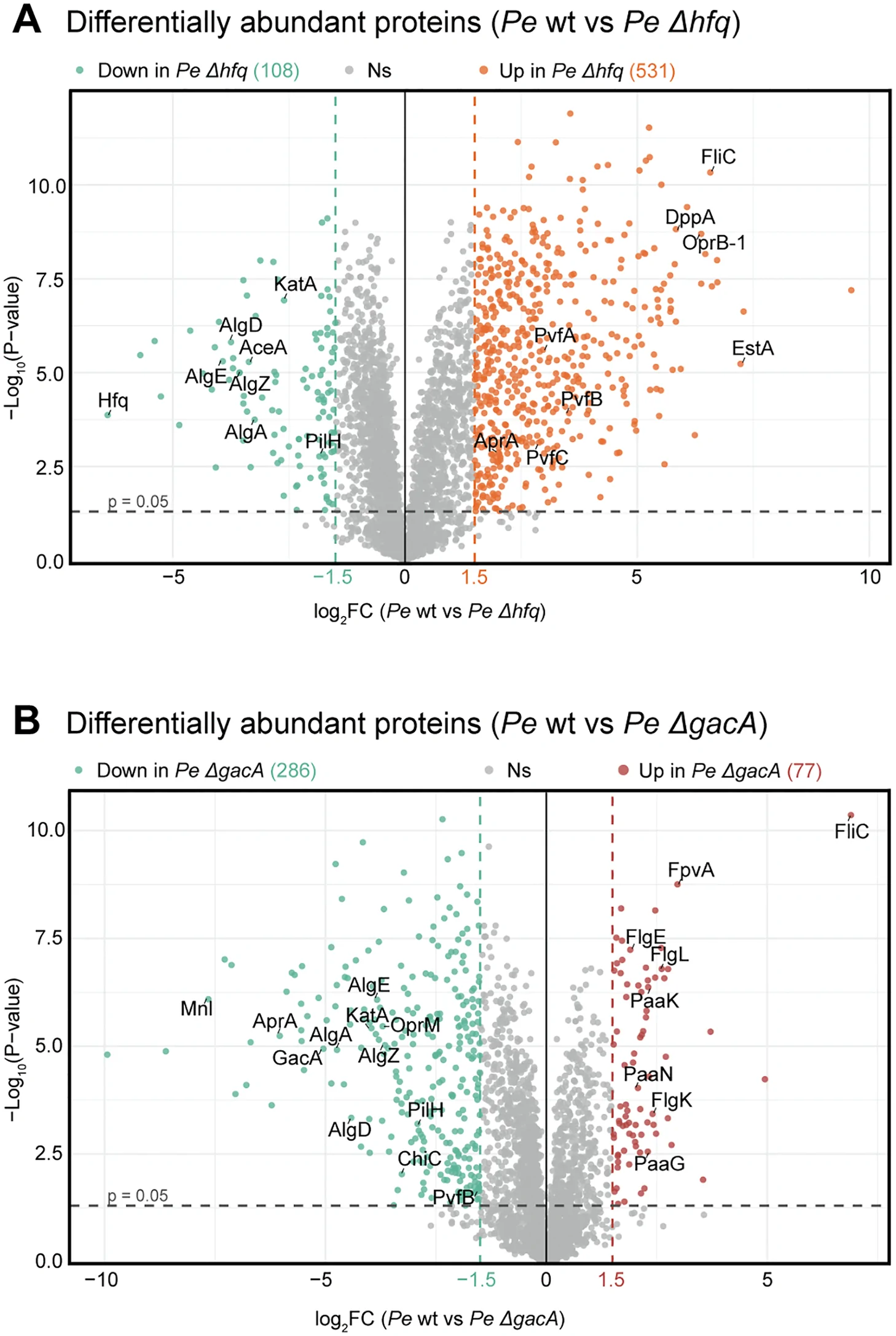
Comparative proteomics identified Hfq- and GacA-regulated proteins. **(A)** Volcano plots of differentially abundant *P. entomophila* proteins (│log2FC│ ≥ 1.5 and padj cut-off 0.05) between *P. entomophila Δhfq* and *P. entomophila* wt. (n = 5 independent samples). **(B)** Volcano plots of differentially abundant *P. entomophila* proteins (│log2FC│ ≥ 1.5 and padj cut-off 0.05) between *P. entomophila ΔgacA* and *P. entomophila* wt. (n = 5 independent samples).

In *ΔgacA* mutant, we found 77 proteins with increased and 286 proteins with decreased abundance as compared to wild-type *P. entomophila* (Fig 3B, S1 Table). Several flagella proteins (FliC, FlgL, FlgK, FlgE) [34] and *ferric pyoverdine receptor FpvA* [35] were among virulence-related factors upregulated in *ΔgacA* mutant. Enrichment analysis identified GO terms related to locomotion (S1C Fig): taxis and flagellar assembly, locomotion and bacterial chemotaxis, chemotaxis signaling, suggesting that deficiency in GacA increases abundance of proteins with a role in motility and chemotaxis. Loss of GacA resulted in a downregulation of a number of virulence factors, including protease AprA, toxin Monalysin, alginate biosynthesis proteins AlgA, AlgD, AlgE, AlgZ, catalase KatA, pilin pilH, chitinase ChiC, *Pseudomonas virulence factor* PvfB (Fig 3B, S1 Table). GO analysis identified an enrichment of proteins related to secretion, carbohydrate metabolism, and biofilm formation among the down-regulated proteins (S1D Fig), suggesting that GacA positively regulates secretion systems and metabolic pathways, promoting bacterial adaptation and stability.

Overall, our proteomic analysis not only determined proteins controlled by the two major regulatory systems, GacA and Hfq, but also identified GacA-controlled candidate factors that may inhibit defecation.

### Monalysin is one of the factors inhibiting defecation

To narrow the list of candidates involved in defecation suppression, we sorted the proteins based on the following assumptions. First, the involved protein should be absent/downregulated in *ΔgacA* mutant, which is partially impaired in defecation blockage, but not in *Δhfq* mutant, which inhibits defecation. Therefore, we performed an additional comparison of the *Δhfq* mutant with the *ΔgacA* mutant to look for additional proteins that are not present in the mutant with impaired defecation blockage (S2A Fig). Second, the involved protein should be present in both *P. entomophila* wild-type and *Δhfq* mutant, which inhibit defecation. Therefore, we examined the overlap of (1) the protein list that showed no significant difference between *P. entomophila* wild-type and *P. entomophila Δhfq* (indicating that it is present at similar levels in both strains (Fig 3A) and the protein list that was present in either (2) *P. entomophila* wild-type or (3) *P. entomophila Δhfq* but not in *P. entomophila ΔgacA*. Using this approach, we identified 146 candidate proteins that meet our criteria (Fig 4A, S2 Table). Most of the proteins are uncharacterized with predicted or hypothetical functions. The limited functional information available for these candidates and the lack of corresponding mutants for many of them limited our ability to formulate specific, experimentally testable hypotheses. We therefore focused on candidates with established or plausible functions and available genetic tools. Among these, we noticed Monalysin as one of the most downregulated proteins in *ΔgacA* mutant (Fig 4B). It caught our attention, as it is a pore-forming toxin previously shown to be involved in *P. entomophila* pathogenesis [24]. Given that pore-forming toxins are known to interfere with gut peristalsis [36], we decided to test the contribution of Monalysin to defecation suppression by *P. entomophila,* using the available *Δmnl* mutant [24]. Although *Δmnl* mutant was still able to reduce defecation frequency in infected flies as compared to uninfected controls, this reduction was not as strong as in flies infected with wild-type *P. entomophila* (Fig 4C, Fig 4C’)*.* Since flies ingested a similar amount of wild-type and *Δmnl* mutant bacteria, the observed defecation differences do not stem from the differential intake of the strains (Fig 4D). Using an ex vivo assay, we confirmed our previous observation that wild-type *P. entomophila* inhibits gut contractions (Fig 4E). Flies infected with *Δmnl* mutant showed significantly higher number of gut contractions compared to flies infected with wild-type bacteria (Fig 4E), suggesting that Monalysin is required for the intestinal peristalsis inhibition and consequent defecation blockage. As a result of its inability to fully suppress peristalsis, the *Δmnl* mutant exhibited a reduced burden in flies (Fig 4F) and reduced virulence (Fig 4G, Fig 4G’). To further reinforce the role of Monalysin in defecation inhibition, we expressed Monalysin in *Escherichia coli* using an arabinose-inducible expression system. Flies fed arabinose-induced *E. coli* exhibited significantly lower defecation frequency than sucrose-fed flies or those fed uninduced *E. coli* (Fig 4H, 4H’). We noticed that even uninduced *E. coli* significantly reduced defecation freaquency when compared to sucrose-fed controls (Fig 4H, 4H’), likely due to a leaky expression of Monalysin. Together, these results support a crucial role of Monalysin in defecation blockage and pathogen persistence.

**Fig 4:**
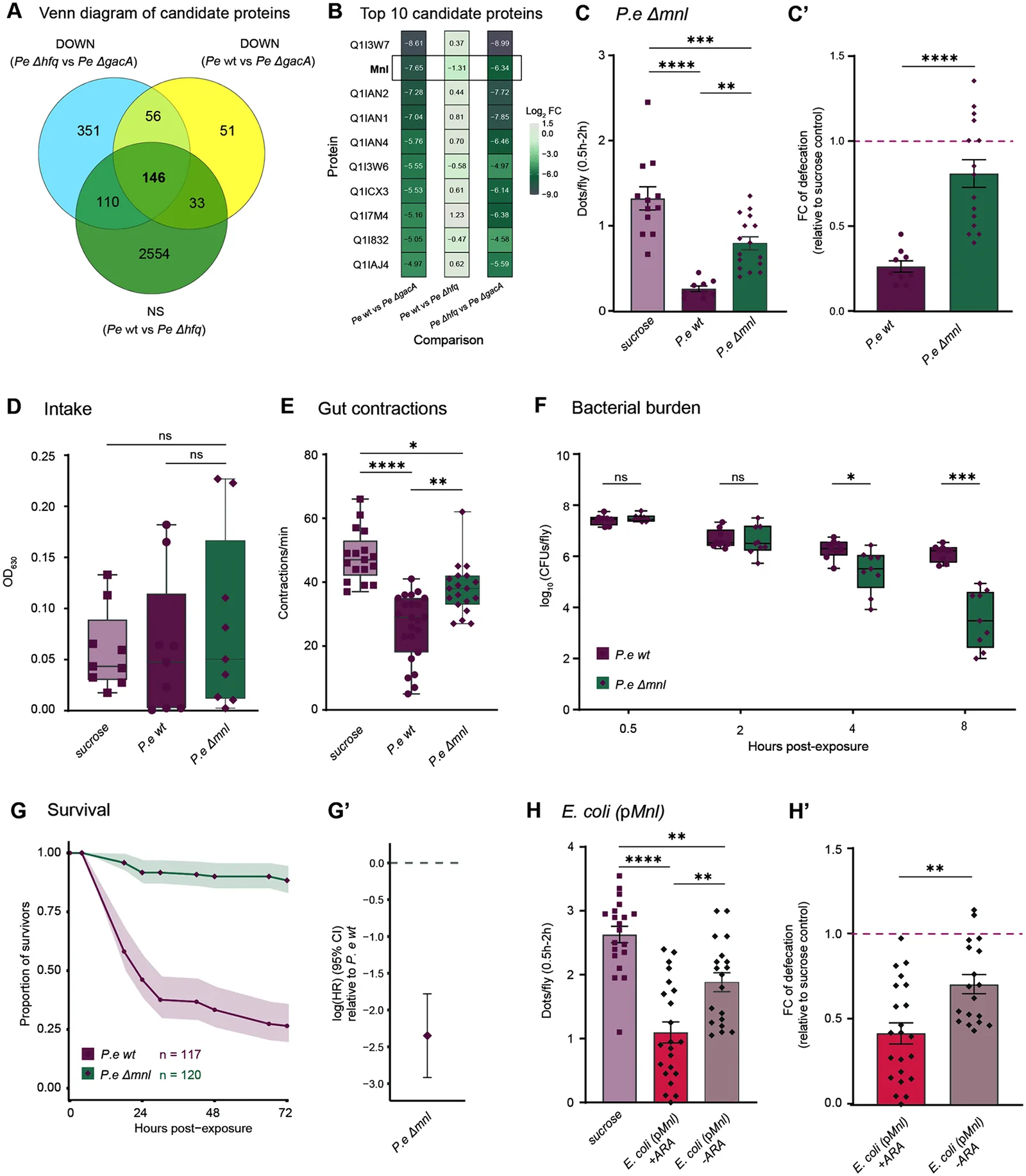
Monalysin is one of the factors inhibiting defecation. **(A)** Venn diagram showing the overlap of candidate proteins identified in three datasets: Down (*P.e* wt vs *P.e ΔgacA*), Down (*P.e Δhfq* vs *P.e ΔgacA*), and Ns (*P.e* wt vs *P.e Δhfq*). **(B)** Heatmap showing abundance difference (log2FC) of top 10 candidate proteins (based on *P.e* wt vs *P.e ΔgacA* log_2_FC values) in three different comparisons: *P.e* wt vs *P.e ΔgacA*, *P.e Δhfq* vs *P.e ΔgacAΔgacA*, and *P.e* wt vs *P.e Δhfq*. Significance (padj < 0.05, |log2FC| > 1.5) indicated by *. The highlightied protein is Monalysin. **(C and C’)** The defecation rate of female *w1118* iso flies measured 0.5 to 2 h after exposure to blue-dyed PBS (control), *P. entomophila* wt or *P. entomophila Δmnl* in 0.5 h protocol. **(C)** Significance by one-way ANOVA with Šídák’s multiple comparisons test. (n (sucrose) = 12, n (*P.e* wt) = 9, n (*P.e Δmnl*) = 16, N (days) = 3) **(C’)** Fold change values were calculated as ratios of the corresponding treatment relative to the mean PBS control from the same biological repeat. Significance by Welch’s unpaired t-test. **(D)** Intake of blue-dyed sucrose (control), *P. entomophila*, or *P. entomophila Δmnl* by female *w1118* iso flies during 0.5 h exposure. Intake was quantified by measuring the OD_600_ of blue dye in flies following exposure. Significance by Kruskal–Wallis test with Dunn’s multiple comparisons test (n = 9 per treatment, N (days) = 3). All indicated pairwise comparisons were not significant. The same data for the sucrose- and *P. entomophila*-fed conditions are also shown in Fig 1E. **(E)** Gut contraction count of female *w1118* iso flies after exposure to sucrose (control), *P. entomophila*, or *P. entomophila Δmnl* using the 1 h ex vivo assay. Significance by Kruskal–Wallis test with Dunn’s multiple comparisons test (n (sucrose) = 17, n (*P.e* wt) = 23, n (*P.e Δmnl*) = 19). **(F)** Bacterial burden of female *w1118* iso flies at different time points after exposure to *P. entomophila* wt or *P. entomophila Δmnl* using the 0.5 h protocol. Each data point represents one biological replicate (pool of five flies). Significance between *P. entomophila* wt and *P. entomophila Δmnl* at each time point was determined by Welch’s unpaired t-tests with Holm–Šídák correction for multiple comparisons (n = 9 per treatment and time point). **(G and G’)** Survival of female *w1118* iso flies following exposure to *P. entomophila* wt or *P. entomophila Δmnl*. **(G)** Kaplan–Meier survival curves with 95% confidence intervals (shaded areas). **(G’)** Hazard ratio (HR) with 95% confidence interval for *P. entomophila Δmnl* relative to *P. entomophila* wt, calculated using a Cox proportional hazards model (HR = 0.095, 95% CI = 0.054–0.169, P < 0.0001). Error bars represent 95% confidence intervals, and the dashed line indicates HR = 1. **(H and H’)** The defecation rate of female *w1118* iso flies measured 0.5 to 2 h after exposure to blue-dyed sucrose (control) or *E. coli* carrying pBAD18-*mnl*, grown in the presence (+Ara) or absence (−Ara) of 0.2% L-arabinose, in the 0.5 h protocol. **(H)** Significance by one-way ANOVA with Šídák’s multiple comparisons test (n (sucrose) = 20, n (*E. coli* pBAD18-*mnl* + Ara) = 22, n (*E. coli* pBAD18-*mnl* − Ara) = 19), N (days) = 5). **(H’)** Fold-change values were calculated relative to the mean sucrose control from the corresponding biological repeat. Significance by Mann-Whitney test.

### *P. entomophila* infection induces Monalysin-dependent alterations in gut morphology and F-actin organization

To determine how *P. entomophila* infection affects intestinal morphology, we examined *Drosophila* guts after infection with wild-type *P. entomophila*, a Monalysin-deficient mutant, or heat-inactivated wild-type bacteria. Guts from sucrose-fed flies served as uninfected controls. DAPI staining revealed marked alterations in the organization and distribution of nuclei in the midgut after infection with wild-type *P. entomophila*, with nuclei appearing less regularly arranged and more heterogeneous in their distribution compared to sucrose-fed controls (Fig 5A-B). These morphological changes were accompanied by a marked reduction and disruption of phalloidin staining compared with sucrose-fed controls, indicating alterations in intestinal F-actin organization. (Fig 5A–B’). In contrast, guts from flies infected with the *Δmnl* strain or exposed to heat-inactivated bacteria displayed overall morphology and phalloidin staining patterns more similar to those of control flies (Fig 5C–D’).

**Fig 5:**
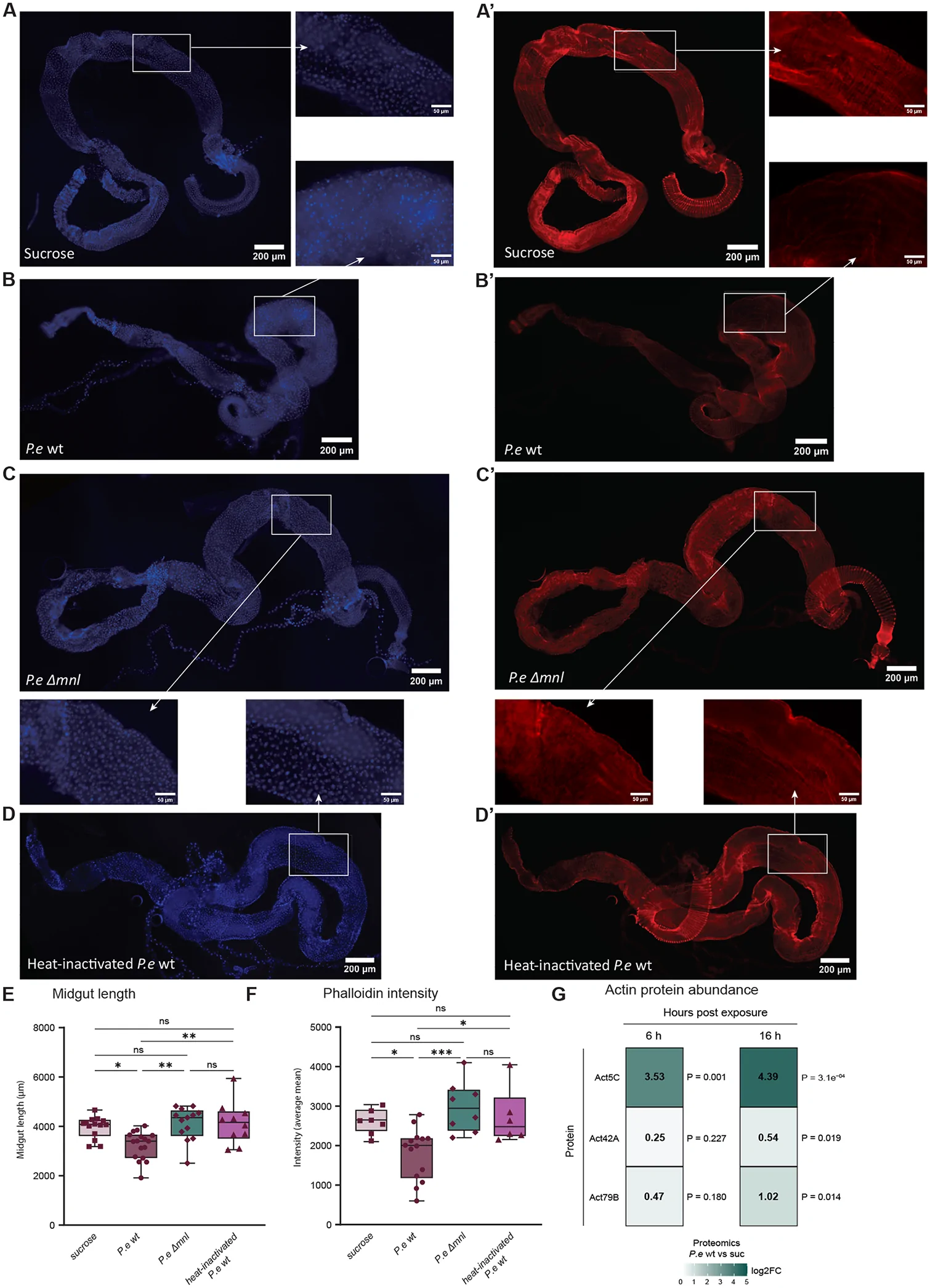
Monalysin-dependent alterations in gut morphology and F-actin organization. **(A and A’)** DAPI (A) and phalloidin (A’) staining of dissected guts from flies treated with 2.5% sucrose after 15 hours. Zoom in images from posterior mitguts (rectangle area) were shown next to the full gut images. **(B and B’)** DAPI (B) and phalloidin (B’) staining of dissected guts from flies infected with wild-type *P. entomophila* after 15 hours. Zoom in images from posterior mitguts (rectangle area) were shown next to the full gut images. **(C and C’)** DAPI (C) and phalloidin (C’) staining of dissected guts from flies infected with *Δmnl* mutant after 15 hours. Zoom in images from posterior mitguts (rectangle area) were shown next to the full gut images. **(D and D’)** DAPI (D) and phalloidin (D’) staining of dissected guts from flies infected with heat-inactivated wild-type *P. entomophila* after 15 hours. Zoom in images from posterior mitguts (rectangle area) were shown next to the full gut images. Scale bar 200 µm for full gut images and 50 µm for zoom in images. **(E)** Midgut length of flies treated with sucrose (control), wild-type *P. entomophila*, *P. entomophila Δmnl*, or heat-inactivated wild-type *P. entomophila*. Significance by one-way ANOVA with Tukey’s multiple comparisons test (n (sucrose) = 14, n (*P.e* wt) = 17, n *(P.e Δmnl*) = 13, n (heat-inactivated *P.e* wt) = 10). **(F)** Phalloidin staining intensity of representative posterior midguts from flies exposed to sucrose (control), wild-type *P. entomophila*, *P. entomophila Δmnl*, or heat-inactivated wild-type *P. entomophila*. Significance by one-way ANOVA with Tukey’s multiple comparisons test (n (sucrose) = 7, n (*P.e* wt) = 14, n (*P.e Δmnl*) = 8, n (heat-inactivated *P.e* wt) = 6). **(G)** Heatmaps showing differences (log2FC, infected guts compared to control guts) in the abundance of selected actin proteins at 6 h and 16 h post-exposure to *P. entomophila*.

Consistent with these qualitative observations, infection with wild-type *P. entomophila* significantly reduced midgut length compared with sucrose-fed controls (Fig 5E). This reduction was largely absent in flies infected with the *Δmnl* strain or exposed to heat-inactivated bacteria, whose midgut lengths were not significantly different from the control condition (Fig 5E). Moreover, wild-type *P. entomophila* infection resulted in a significant reduction in phalloidin staining intensity, indicative of alterations in intestinal F-actin organization or abundance (Fig 5F). Phalloidin staining intensity was significantly higher after infection with the *Δmnl* mutant than following infection with wild-type bacteria and was not significantly different from the sucrose control (Fig 5F). Similarly, heat-inactivated bacteria did not significantly reduce phalloidin staining compared to the control (Fig 5F).

Together, these results indicate that alive *P. entomophila* induces changes in intestinal morphology and F-actin organization and that both phenotypes are dependent on Monalysin. The absence of comparable changes following exposure to heat-inactivated bacteria further suggests that these effects require bacterial activity rather than exposure to bacterial components alone.

To determine whether the reduced F-actin signal after infection was associated with changes in total actin abundance, we examined the abundance of three actin proteins expressed in the adult *Drosophila* gut [37] in our previously published proteomic dataset [23]. While Act5C abundance increased following *P. entomophila* infection, Act42A and Act79B did not change significantly at either 6 or 16 h post infection (Fig 5G). Thus, the reduction in F-actin staining was not accompanied by a general decrease in actin protein abundance, suggesting that infection may affect actin polymerization and/or cytoskeletal organization.

### Monalysin is conserved among *P. entomophila* strains and related bacterial species

To place Monalysin in an evolutionary context, we compared the Monalysin protein sequence from *P. entomophila* with homologous proteins identified in additional *P. entomophila* strains and related bacterial isolates. Phylogenetic analysis revealed that Monalysin homologs from the three *P. entomophila* strains clustered together, indicating conservation of the protein within this species (Fig 6). Monalysin-like proteins were also identified in several other *Pseudomonas* isolates, as well as in more distantly related bacterial genera, including *Cystobacter* and *Melittangium* (Fig 6). Thus, Monalysin-like proteins are not restricted to the *P. entomophila* lineage but occur across a broader range of bacteria. The conservation of Monalysin-like proteins across multiple bacterial lineages raises the possibility that manipulation of intestinal physiology by pore-forming toxins represents a more broadly conserved strategy during host colonization.

**Fig 6:**
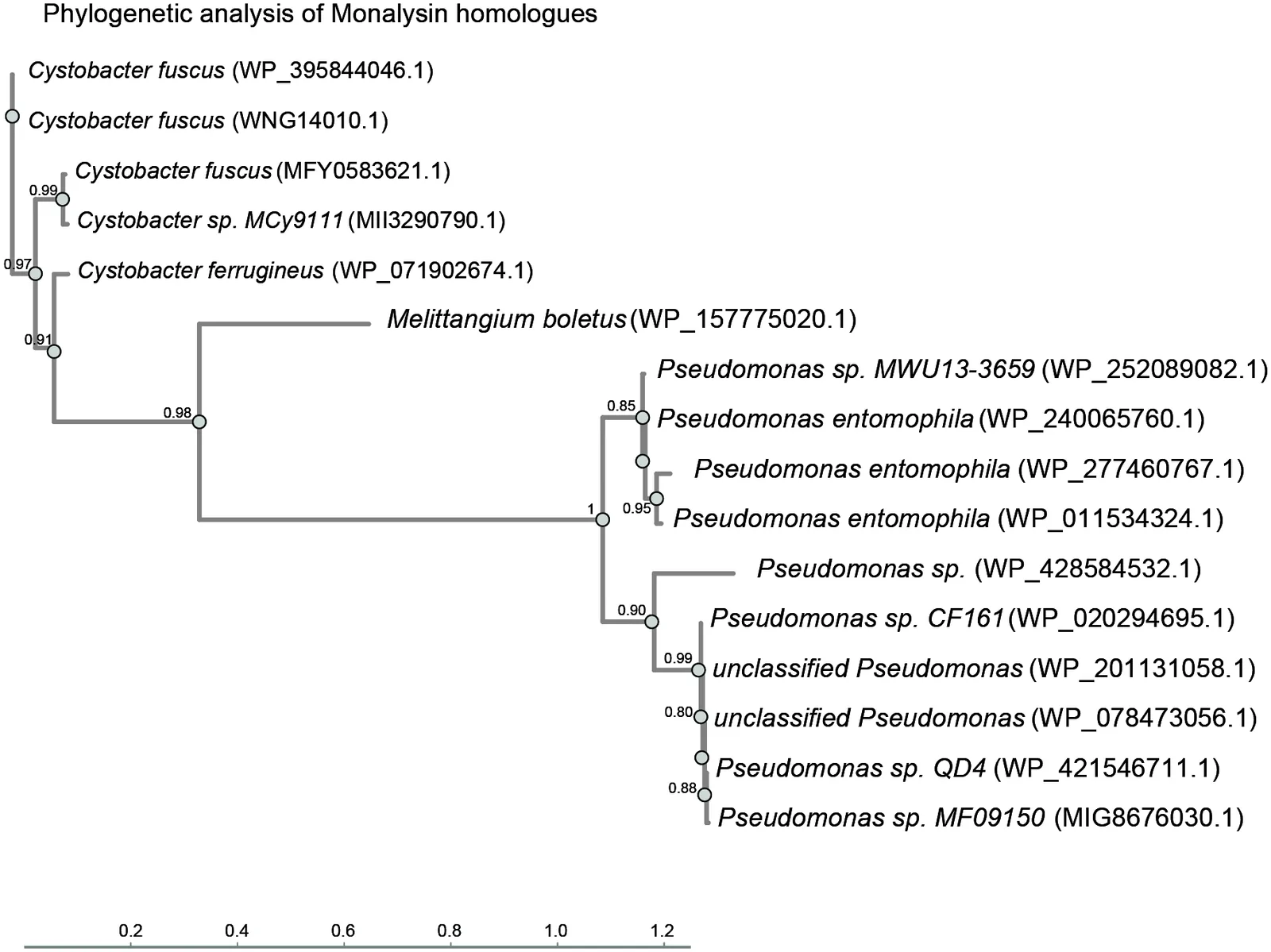
Phylogenetic analysis of Monalysin homologues. Phylogenetic analysis of Monalysin homologues identified by BLASTP. Protein sequences were aligned using Clustal Omega, and a maximum-likelihood phylogenetic tree was inferred using PhyML with the WAG + G + I + F substitution model selected by SMS. Numbers at internal nodes indicate SH-like aLRT branch-support values (values ≥ 0.80 shown). Accession numbers are indicated in parentheses.

## Discussion

Our study identified a previously unrecognized bacterial factor contributing to infection-induced suppression of defecation frequency in *Drosophila melanogaster*. While several microbes have been shown to accelerate gut motility, *P. entomophila* induces the opposite effect — a marked reduction in defecation, particularly in females. We show that this phenotype does not depend solely on bacterial viability but rather on a secreted, thermosensitive protein whose production is partially regulated by the GacS/GasA system, and not by the RNA chaperone Hfq.

By combining defecation assays and proteomic analyses, we identified Monalysin as one of the bacterial factors required for suppressing defecation and bacterial clearance. Monalysin is a pore-forming toxin previously shown to be responsible for damage of host intestinal cells [24]. Monalysin also contributes to translation blockage [38] and thinning of the intestinal epithelium associated with *P. entomophila* infection [39]. Our findings expand the role of Monalysin in *P. entomophila* pathogenicity by demonstrating that Monalysin also perturbs gut homeostasis in a way that reduces intestinal transit. The mechanism by which Monalysin causes rapid inhibition of defecation remains to be explored. It could be that intestinal damage inflicted by Monalysin is sufficient to stop peristalsis, as has been suggested for Cry toxins [40,41]. Given its pore-forming activity, Monalysin could perturb ion homeostasis in intestinal tissues and thereby interfere directly or indirectly with the signaling required for visceral muscle contraction. Alternatively, Monalysin-induced epithelial damage could alter pathways regulating gut motility, including TRPA1-dependent signaling. But distinguishing between these mechanisms will require further investigation.

However, the fact that the *Δmnl* mutant still reduced defecation frequency, although not as strongly as wild-type bacteria, suggests that additional factors besides Monalysin are required to block intestinal peristalsis. Our proteomic comparisons uncovered additional GacA-dependent proteins that may contribute to the phenotype. Among the top 10 most downregulated proreins in *ΔgacA* mutant, there are uncharacterized proteins (Q1IAN1, Q1IAN2); putative metabolic enzymes (Q1IAN4 Putative oxidoreductase, NAD-binding Ros family, Q1ICX3 CMP/dCMP-type deaminase domain-containing protein, Q1IAJ4 Putative inosine-5’-monophosphate dehydrogenase); inclusion body proteins (Q1I3W6, Q1I3W7); and putative methyl-accepting chemotaxis transducer (Q1I7M4). Analysis of inclusion body proteins in *Xenorhabdus nematophila* showed that they are not required for virulence or colonization of insect hosts [42]. Given the prominent role of chemotaxis in bacterial virulence [43], the methyl-accepting chemotaxis transducer (Q1I7M4) is an attractive candidate for assessing its contribution to defecation blockage. Our work, by identifying these proteins provides a starting point for future studies aiming to dissect the bacterial strategies that manipulate host intestinal peristalsis.

Previously, *P. entomophila* was shown to cause food intake cessation in *Drosophila* larvae [25]. Our results provide indirect evidence that Monalysin might cause this effect by inhibiting intestinal peristalsis. Another *Drosophila* pathogen, *Ecc15*, requires *evf* (*Erwinia* virulence factor) gene for bacterial persistence in the larval gut. Although the mechanism of action of *evf* is unknown, *evf* was proposed to inhibit gut peristaltic movements and, hence, facilitate bacterial persistence [44,45]. Pore-forming Cry toxins produced by insecticidal *Bacillus thuringiensis* strains have also been implicated in feeding cessation and suppression of peristalsis [40,41]. Hence, abrogation of peristalsis is frequently observed in insects during intestinal infections. On the one hand, inhibiting peristalsis would allow the host to reduce the amount of ingested pathogen, repair the midgut epithelium, and reduce pathogen transmission via the fecal route. Additionally, trapping microbes in a specific gut region can help the host kill pathogens with antimicrobial peptides [46]. On the other hand, inhibition of peristalsis would prevent pathogen eviction from intestine via defecation, facilitating bacterial persistence [23,46]. The facts that pathogens have developed strategies to abrogate peristalsis and hosts with defects in intestinal transit suffer more from infections [36,47], suggest that pathogens benefit from inhibiting gut transit. However, our results show that virulence and inhibition of peristalsis are not necessarily linked. The *Δhfq* mutant, despite being avirulent, still inhibits defecation. This indicates that defecation blockage is not directly associated with virulence or host killing but may represent an adaptive bacterial strategy. Reduced gut motility could prolong bacterial presence in the intestine, enhance colonization, or alter the gut environment in ways favorable for the pathogen. Our findings also open broader questions about how non-pathogenic bacteria affect intestinal peristalsis and whether inhibition of defecation is inherently detrimental to the host. In some cases, reduced motility might limit bacterial dissemination or moderate immune activation, while in others it could promote bacterial retention and chronic infection. The presence of this phenotype in avirulent strains suggests that modulation of gut transit may be a widespread microbial strategy rather than a purely pathogenic one.

Altogether, our findings describe a bacterial factor that suppresses the host's intestinal peristalsis and the ability to evict bacteria from the gut via defecation. Further elucidation of the underlying mechanisms will not only broaden our understanding of pathogen strategies used to subvert host defences but could also open the way to new methods of insect pest control.

## Methods

### Arrive protocol

The ARRIVE 2.0 guidelines were followed in planning, conducting and reporting *in vivo* experiments [48].

### *Drosophila* husbandry

*Drosophila* stocks were raised in a light:dark cycle (12 h:12 h) at 25°C, on a standard cornmeal/agar medium (6.2 g agar, 58.8 g cornmeal, 58.8 g inactivated dried yeast, 26.5 ml of a 10% solution of methyl-paraben in 85% ethanol, 60 ml fruit juice, 4.8 ml 99% propionic acid for 1 L). For maintaining flies, flies were transferred to fresh vials every 2–3 days, and fly density was kept to a maximum of 15 flies per vial. At day 2 after eclosion, all emerged adults were transferred to new vials where they were left to mate for additional 2 days. Female flies were then sorted using CO_2_ in vials with 10 or 20 flies per vial the evening before experiment. Therefore, 5–7 days old mated female *w1118* iso flies were used in all experiments.

### Bacterial strains

*Pseudomonas entomophila* L48 strain [22], *P. entomophila ΔgacA* mutant [25], and *P. entomophila Δmnl* [24] were obtained from Bruno Lemaitre’s lab. Previously published *P. entomophila Δhfq* mutant [49] was kindly provided by Dr. Edna Bode (Max Planck Institute for Terrestrial Microbiology).

### Preparation and treatments of bacteria and bacterial supernatant

*P. entomophila* strains utilized in this study were grown directly from frozen 50% glycerol stocks. Briefly, 10 µL of the stocks was incubated in 20 mL of LB medium in a 50 mL flask overnight (∼16 h, 29°C, 175 rpm). The following day, the overnight culture was diluted 1:16 in 150 mL of LB medium and incubated in 500 mL flasks under the same conditions for a minimum of an additional 24 hours. Before the defecation experiment, 45 mL of bacterial culture was centrifuged at 3,500 × g for 15 min at 4 °C using 50 mL screw-top tubes (114 × 28 mm, PP; Sarstedt AG & Co.).

(a) For preparing bacterial suspensions, the supernatant was discarded and the bacterial pellets were resuspended in PBS. The final concentration was adjusted to an optical density (OD600) of 200 in PBS.
(b) For preparing cell-free supernatant, after centrifugation of bacterial culture, 2 mL of the supernatant (taken from the top) was filtered using a Millex filter unit (0.22 µm, sterile; Merck Millipore).
(c) For heating treatments, 500 µL of bacterial suspension or supernatant was placed in a 1.5 mL Eppendorf vial, heated for 15 min at 95°C (Eppendorf ThermoMixer), and then left to cool for 15 min at room temperature before next steps.
(d) For proteinase K treatment, the supernatant was mixed with either control (same volume of ddH_2_O) or proteinase K (AM2548, Ambion, final concentration: 1 mg/mL) and incubated for 30 min at 37 °C (Eppendorf ThermoMixer)), and then left to cool for 15 min at room temperature before next steps.

### Quantifying defecation in adult flies

For quantifying defecation, the bacterial suspension or supernatant (described above) were mixed in a 1:1 ratio with a 5% sucrose solution containing 1% blue dye (Food Blue No. 1, TCI) (referred to as blue mixture) as described previously [18]. Prior to the defecation experiment, 150 µL of blue mixture was pipetted into vials containing standard food and filter papers (referred to as infection vials). Before transferring mated flies to the infection vials, the flies were kept in empty vials for 2 hours at 29°C (dry starvation). Flies were kept in the infection vials for 0.5 h before being transferred to the vial containing conventional non-colored food for an additional 1.5 hours. Defecation quantity was measured by counting ‘defecation dots’ left dried on the inner wall of vials and normalized per number of flies in vial. All experiments were performed at 29°C and at least three times (different days), with a total of at least 9 independent vials per condition, each containing 10 or 20 flies.

### Infection and pathogen load assays

Fly oral infections were carried out at 29°C on 5–7 days old mated female *w1118* iso flies as previously described [23]. All the infections, except when specified, were carried out with bacterial preparation adjusted to an OD600  =  100 (OD600 = 200 mixed 1:1 with 5% sucrose) which corresponds to 8.7-9.8E10 CFUs per ml. All *P. entomophila* strains used in this study showed comparable CFUs counts for a given OD600. 150 µL of pathogen mixture was deposited on a filter disk that completely covered the surface of standard fly medium. Before transferring flies to the infection vials, the flies were kept in empty vials for 2 hours at 29°C (dry starvation). Flies were incubated for one day at 29°C on the contaminated filter, after which they were transferred to fresh vials without living yeast. All experiments were performed at least twice on different days with at least two vials per day, each containing 20 flies.

To measure bacterial load in flies, 5 female flies were collected at 0.5, 2, 4 and 6 hours post infection. The samples were first surface-sterilized in 70% ethanol for 20 seconds, with three washes and one in sterile PBS for 30 seconds. Then, the samples were homogenized in 500 μL of sterile PBS for 30 seconds at 6000 rpm using a Precellys 24 instrument (Bertin Technologies, France). Serial 10-fold dilutions ranging from 10^−^^1^ to 10^−6^ were made, and 10 μL of each dilution with 3 replicates were plated on LB plates. After an overnight incubation (proxy 18 h) at 30°C, the number of colonies were counted. Flies fed with sucrose were processed in parallel with infected flies as a control. Since the control plates were negative for bacterial colonies, it was assumed that all colonies that grew on plates from infected flies were *P. entomophila.*

### Ex vivo quantification of gut contractions

Guts were carefully dissected directly in Schneider’s medium, transferred onto microscope slide type with concave indentions (VWR, LOT 0180) that contained 100 μl Schneider’s medium (room temperature). Samples were covered with r coverslips (Roth, 24 × 24 mm, #1, Art. No. 875). Videos were acquired on a Leica Thunder microscope in dark field mode using a 5 × /0.15 NA dry objective. A total recording time of 1 minute where an image was taken every 200 ms. Gut contractions were counted manually. Any visible contraction in any region of the gut was considered as a contraction readout. Each experiment was performed in at least three independent biological repeats, with a minimum of two guts imaged per repeat.

### Food intake quantification

The amount of ingested food was quantified by including blue dye (Food Blue No. 1, TCI) in the infection mixture modified from a previous method [23]. *P. entomophila* was mixed at a 1:1 ratio with a 5% sucrose solution containing 1% blue dye. After 0.5 h, feeding was interrupted and five flies were transferred to tubes containing glass beads and 500 μL of 1x PBS with 0.1% Triton X-100 (PBST). The samples were then homogenized using a Precellys homogenizer (three cycles of 30 seconds at 7,200 rpm) and then centrifuged twice (2 mins, maximum speed). Then, 300 μL of the supernatant was then transferred to a cuvette containing 700 μL of water. Blue dye consumption was quantified by measuring absorbance at 630 nm using an Ultrospec 2100 pro UV/Vis Spectrometer (Amersham Biosciences).

### Imaging methods

#### Sample preparation and microscopy.

Three to eight days old conventional female *w1118* iso flies were collected, and infected with *P. entomophila*, *Δmnl* mutant, and heat-inactivated *P. entomophila* for 15 hours. Sucrose was used as control. Fly guts were dissected and immediately fixed in 1 × PBS containing 4% paraformaldehyde for 30 min at room temperature. Samples were then rinsed three times with 1 × PBS and incubated for 30 min in 500 µl blocking buffer (1% bovine serum albumin, and 0.1% Tween-20 dissolved in PBS). Samples were then incubated with far-red phalloidin Alexa Fluor 647 (Sigma-Aldrich, final dilution 1:400) in PBST for 60 min at room temperature in dark. Samples were washed with PBST three times. One µl of DAPI (5 mg/ml; final dilution 1:500) was then added to 500 µl PBST, and samples were additionally incubated for 10 min at room temperature in the dark. Guts were then mounted in Mowiol 8% (Sigma-Aldrich). Images were acquired using a Leica Thunder widefield fluorescence microscope. Z-stacks of 80–100 µm were collected, and 10 z-steps were applied for each gut.

#### Gut length quantification.

Gut length was quantified using Fiji (ImageJ, NIH). Raw fluorescence images were calibrated using the microscope pixel size to convert pixel measurements to micrometers (μm). The centerline of each dissected gut was manually traced from the anterior midgut to the posterior midgut using the Freehand Line tool, following the longitudinal axis of the gut lumen. Gut length was recorded in micrometers using the *Measure* function. The same anatomical landmarks and tracing criteria were applied to all samples.

#### Phalloidin staining intensity quantification.

Adult *Drosophila* midguts stained with phalloidin were imaged under identical acquisition settings for all samples. Z-stack images (10 optical sections) were acquired for each gut. Raw 16-bit images were analyzed in Fiji without modification of pixel intensities. For each optical section, the same anatomical region of the middle midgut epithelium was manually outlined using the Polygon selections tool, and the mean gray value was measured. Background fluorescence was measured from a nearby cell-free region in the same optical section and subtracted from the mean gray value. The background-corrected mean fluorescence intensity was calculated for each optical section, and the average value across all z-slices was used as the fluorescence intensity for one gut. Each gut was treated as one biological replicate for statistical analysis.

### Cloning and expression of *mnl*

The *mnl* gene from *Pseudomonas entomophila* was amplified by PCR using the primers mnlF EcoRI (5′-GCCGGAATTCacgagggaaggacgatgacgat-3′) and mnlR HindIII (5′-GCCCAAGCTTtcaataacggcgatgaggatcg-3′), containing EcoRI and HindIII restriction sites, respectively. The PCR product was cloned into the corresponding EcoRI and HindIII sites of the arabinose-inducible pBAD18 expression vector. The resulting pBAD18-*mnl* construct was introduced into *E. coli* TOP10 cells, which were used for both cloning and heterologous expression of *mnl*. *E. coli* was grown overnight in LB at 37°C and diluted 1:100 the following day into fresh LB supplemented with 50 µg/mL ampicillin. Upon reaching an OD600 of approximately 0.5, *mnl* expression was induced by addition of L-arabinose to a final concentration of 0.2%, followed by incubation at 37°C for 4 h. An identically treated culture without L-arabinose served as the uninduced control. Following induction, bacterial cultures were adjusted to an OD600 = 100 and mixed 1:1 with 5% sucrose containing 1% blue dye. The defecation assay was subsequently performed as described above.

### *P. entomophila* proteome comparison

Using 1.5 mL Eppendorf tubes, 1 mL of bacterial culture (OD600 = 1, adjusted in LB) was centrifuged at 3,500 × g for 15 min at 4 °C, after which 950 µL of the supernatant was discarded. The pellet was resuspended, and 50 µL of the suspension was transferred to tubes containing glass beads and 500 µL of protein extraction buffer (100 mM Tris-Cl, pH 8.0, 2% SDS, protease inhibitor (cOmplete, Roche, 1 tablet per 4 mL of buffer)) on ice. Following incubation (95°C for 2 minutes) and homogenization (6000 rpm for 30 seconds, Precellys homogenizer), samples were centrifuged (max speed, 10 min, 4°C). Supernatant was collected into Eppendorf tubes. Protein concentrations were measured by the Pierce BCA Protein Assay Kit (Thermo Fisher Scientific Pierce BCA Protein Assay Kit Catalog Numbers 23225 and 23227) according to the manufacturer’s protocol. Briefly, 10 μL of samples were incubated with 200 μL of working reagent and incubated for 30 min at 37°C. Absorbance measurements (in duplicates) were performed at 562 nm using a plate reader Infinite M Plex Microplate Reader (Tecan). Samples were stored at -80°C before being analyzed.

(a) **Sample preparation (SP3)**

All samples were subjected to SP3 sample preparation protocol [50]. Briefly, 10 μg protein from each sample was combined with 4 × lysis buffer (50 mM HEPES [pH 8; VWR], 4% SDS (vol/vol) (Applichem), 160 mM 2-chloroacetamide (CAA), 40 mM Bond-Breaker Tris(2-carboxyethyl)phosphine hydrochloride [TCEP] solution [Thermo Fisher Scientific]) to achive a final concentration of 1 × . Samples were incubated for 5 min at 95°C, then cooled to room temperature. Benzonase nuclease (Merck; 0.5 units per μg protein) was then added, and samples were incubated for 30 min at 37°C with shaking at 500 rpm.

Hydrophilic and hydrophobic carboxyl-coated Sera-Mag SpeedBeads (catalog no. 45152105050250 and 65152105050250, respectively; GE Healthcare) were mixed at a 1:1 ratio and added at a bead-to-protein ratio of 10:1 (w/w). Samples were pipette mixed and anhydrous acetonitrile (Thermo Fisher Scientific) was added to a final concentration of 70% (v/v), and samples were incubated for 20 min at room temperature to promote protein binding to the beads. Following this, beads were collected using a magnetic stand for 5 min, and the supernatant was discarded. The beads were washed three times with 80% ethanol (v/v), with each wash performed for 3 min, and subsequently air-dried for 10 min at room temperature.

The beads were resuspended in 30 μL of 50 mM triethylammonium bicarbonate containing trypsin (Serva) and LysC (Wako) at an enzyme-to-protein ratio of 1:50 (w/w). Protease digestion was performed for 14 h at 37°C in a PCR cycler. After digestion, the beads were collected on a magnetic stand for 5 min, and the peptide-containing supernatant was collected. The peptide supernatants were then acidified with 2% ACN and 0.1% trifluoroacetic acid (TFA).

Label-free DIA proteomics was performed using an Orbitrap Exploris 480 mass spectrometer (Thermo Scientific) coupled to a 3000 RSLC nano UPLC system (Thermo Scientific) as described earlier, with some modifications [51]. A total of 500 ng of peptides were loaded onto a PepMap C18 trap cartridge (300 μm inner diameter × 5 mm; Thermo Scientific) using 2% acetonitrile and 0.1% TFA at 20 μL/min and subsequently separated on a 25 cm PepSep C18 analytical column (75 μm inner diameter, 1.5 μm particle size) maintained at 50°C.

Chromatographic separation was performed at 250 nL/min over 120 min. The mobile phases consisted of 0.1% formic acid in water (solvent A) and 0.1% formic acid in 80% acetonitrile (solvent B). Solvent B was increased from 3% to 30% over 70.5 min and subsequently from 30% to 45% over 13 min, followed by column washing and re-equilibration.

DIA acquisition was performed with MS1 and MS2 resolutions of 60,000 and 15,000, respectively. The MS1 acquisition range was 350–1250 m/z, and MS2 spectra were acquired over 110–1600 m/z. The normalized AGC target was set to 1000%, with a maximum injection time of 50 ms and a normalized collision energy of 30. Precursor ions were acquired using 10 m/z isolation windows with optimized window placement spanning 390.4273–1210.8002 m/z.

Raw data analysis was performed using Spectronaut (Biognosys AG, Zurich, Switzerland) version 20.2.250922.92449 in directDIA+ deep mode with reviewed UniProt databases (*Pseudomonas entomophila* (strain L48) - Proteome ID UP000000658, 5,126 entries). Methionine oxidation and protein N-terminal acetylation were specified as variable modifications, whereas carbamidomethylation of cysteine residues was used as a fixed modification. The false discovery rate (FDR) was set to 0.01 at the peptide-spectrum match, peptide, and protein levels. All tolerances were set to dynamic for Pulsar searches.

Downstream differential analysis was performed using Perseus version 2.0.7.0. Differences in proteomic profiles between bacterial strains were considered significant at an adjusted p value <0.05 and an absolute log2 fold change >1.5. Protein-set overlap was assessed using Venny version 2.1 (https://bioinfogp.cnb.csic.es/tools/venny/index.html). Data visualization was performed using the R package ggplot2.

GO enrichment analysis was performed by copying the lists of significantly upregulated or downregulated proteins from each dataset into ShinyGO v0.85.1 [52]. The species was specified as *Pseudomonas entomophila* (taxonomy ID: 384676) using the STRING-db annotation (STRING v12.0). The background list consisted of all proteins detected in the corresponding comparison. Analyses were performed using default settings (FDR cutoff of 0.05, 20 pathways to display, and the option to remove redundancy.

The mass spectrometry proteomics data have been deposited to the ProteomeXchange Consortium via the PRIDE partner repository [53] with the dataset identifier PXD071560.

### Phylogenetic analysis

The amino acid sequence of Monalysin from *Pseudomonas entomophila* (WP_011534324.1) was used as a query for BLASTP against the NCBI ClusteredNR (nr_cluster_seq) protein database. Hits with ≥50% sequence identity and ≥80% query coverage were retained, resulting in 17 protein sequences, which were retrieved in FASTA format. The truncated Monalysin sequence corresponding to the experimentally determined structure (PDB: 4MJT_A) was excluded from the phylogenetic analysis. Protein sequences were aligned using Clustal Omega through the EMBL-EBI Job Dispatcher with default parameters [54]. The resulting multiple sequence alignment, comprising 16 sequences and 302 aligned positions, was used for maximum-likelihood phylogenetic analysis with PhyML through NGPhylogeny.fr [55]. Smart Model Selection (SMS) was used to determine the best-fitting amino acid substitution model according to the Akaike Information Criterion (AIC). Maximum-likelihood tree inference was performed using PhyML with the selected WAG + G + I + F model and otherwise standard settings, with SPR topology search and SH-like aLRT branch support. SH-like aLRT support values ≥0.80 are displayed at internal nodes in the final tree.

### Quantification and statistical analysis

Statistical parameters and tests are shown in the figure legends. No formal randomization method was used, but to reduce the potential bias, control and treatment groups were processed in parallel, and sample sizes were balanced. Blinding was not implemented in this study. No data were excluded from analysis. Data analyses and visualization of defecation data were performed using GraphPad Prism 10 software. Proteomics visualization was performed with the R packages ggplot2, dplyr, and tidyverse. Data distributions were assessed using the Shapiro–Wilk test. Depending on data distribution, statistical significance was assessed using one-way ANOVA, Welch’s unpaired t-test, Kruskal–Wallis test, or Mann–Whitney test, with Šídák’s, Tukey’s, Dunn’s, or Holm–Šídák multiple-comparisons correction where appropriate, as indicated in the figure legends. Significance was set at p < 0.05, with data presented as mean ± SEM. Significance: *p < 0.05, **p < 0.01, ***p < 0.001, ****p < 0.0001.

## Supporting information

S1 Fig Gene Ontology (GO) enrichment analysis of Hfq- and GacA-regulated proteins in *P. entomophila.* **(A and B)** GO enrichment analysis of proteins differentially abundant in *P. entomophila Δhfq* relative to *P. entomophila* wt. Panels show the top enriched GO terms (p < 0.05) are shown for **(A)** upregulated proteins and **(B)** downregulated proteins identified in the *P. entomophila Δhfq*. **(C and D)** GO enrichment analysis of proteins differentially abundant in *P. entomophila ΔgacA* relative to *P. entomophila* wt. Panels show the top enriched GO terms (p < 0.05) are shown for **(C)** upregulated proteins and **(D)** downregulated proteins identified in the *P. entomophila ΔgacA*.
(TIF)

S2 Fig Differences between *P. entomophila Δhfq* and *P. entomophila ΔgacA* proteomic profiles. **(A)** Volcano plots of differentially abundant *P. entomophila* proteins (│log2FC│ ≥ 1.5 and padj cut-off 0.05) between *P. entomophila Δhfq* and *P. entomophila ΔgacA*. (n = 5 independent samples).
(TIF)

S1 Table Excel tables listing *P. entomophila* proteins with differential abundance between strains. (XLSX)

S2 Table List of 146 candidates. (XLSX)
