# Supplementary material for: A pore-forming toxin monalysin contributes to infection-induced suppression of defecation in female Drosophila
Source: PLoS Pathog. 2026 Oct 8;22(10):e1014696. doi: 10.1371/journal.ppat.1014696 (PMC13671641; doi:10.1371/journal.ppat.1014696)
Supplement: Supplementary file 2 [file ppat.1014696.s006.docx]

**Point by point responses to the referees comments on** **Plos Pathogens manuscript PPATHOGENS-D-26-02015 entitled “A Pore-Forming Toxin Monalysin Contributes to Infection-Induced Suppression of Defecation in Female Drosophila”**

We thank the reviewers and editors for their valuable suggestions and favorable evaluation of our work. We addressed the remaining minor suggestions of the reviewer 1 and formatted the manuscript to meet the journal requirements.

Reviewers' Comments:

Part I - Summary

Please use this section to discuss strengths/weaknesses of study, novelty/significance, general execution and scholarship.

Reviewer #1: From the submission in Review Commons, all my comments were answered, tested and discussed, thanks a lot.

The manuscript is really improved this way.

Reviewer #2: Defecation is a mechanism that contributes to an organism's defense against ingested pathogens. Accelerated defecation favors the elimination pathogens, whereas slowed defecation promotes their persistence.

The authors identified the Monalysin toxin from Pseudomonas entomophila as a key factor in blocking defecation. Their experiments clearly demonstrate the role of this toxin in inhibiting muscle contractions and the persistence of bacteria, which is associated with higher mortality.

The authors have fully addressed the questions I had raised. They also included the requested experiments. Considering the additional experiments, the supplementary information provided, and the revisions made to address the queries from the other two reviewers, I believe this work deserves to be published in PLOS Pathogens.

Reviewer #3: I thank the authors for addressing my comments and for the additional analyses included in the revised manuscript. In particular, the authors have clarified the rationale for selecting Monalysin from the proteomic candidates, expanded the discussion of other potential factors, added data addressing the physiological relevance of the phenotype, clarified the infection conditions and CFU amounts, and provided a comparative phylogenetic analysis of Monalysin homologues.

The revisions adequately address my concerns, and the manuscript has been substantially improved. I am satisfied with the authors’ responses and have no further major comments.

I therefore support publication of the manuscript in its revised form.

R. We thank all reviewers for their positive feedback.

Part II – Major Issues: Key Experiments Required for Acceptance Please use this section to detail the key new experiments or modifications of existing experiments that should be absolutely required to validate study conclusions.

Generally, there should be no more than 3 such required experiments or major modifications for a "Major Revision" recommendation. If more than 3 experiments are necessary to validate the study conclusions, then you are encouraged to recommend "Reject".

Reviewer #1: (No Response)

Reviewer #2: None. The authors have addressed all of my previous questions and suggestions.

Reviewer #3: (No Response)

Part III – Minor Issues: Editorial and Data Presentation Modifications Please use this section for editorial suggestions as well as relatively minor modifications of existing data that would enhance clarity.

Reviewer #1: *P.e. and F-actin in the guts:

RT-qPCR or Western blot quantif of F-actin in guts from sucrose fed vs guts from P.e. fed animals.

>Will not change the conclusions, but may pave the way to study this extremely strong phenotype.

R. We thank the reviewer for this suggestion. To address this comment and to determine whether the reduced F-actin signal following P. entomophila infection is associated with changes in total actin protein abundance, we examined the abundance of several actin proteins relevant to the Drosophila gut in our proteomic dataset from PNAS study. Interestingly, Act5C abundance was significantly increased following infection, whereas Act42A and Act79B abundance did not change significantly at either 6 or 16 h post infection (new Figure 5G). Thus, the pronounced reduction in phalloidin staining is not accompanied by a general reduction in the abundance of these gut-relevant actin proteins. These results suggest that the reduced F-actin signal may instead reflect changes in actin polymerization and/or cytoskeletal organization during infection. We agree that this striking phenotype warrants further investigation.

*1E and 4D, food intake legend: please indicate that the set of data is the same in both figures.

R. Indicated.

*Please mention directly in the text why 29°c was choosen while it is a stressful temparature for the flies.

R. Mentioned.

Reviewer #2: None. The authors have addressed all of my previous questions and suggestions.

Reviewer #3: (No Response)
