# Supplementary material for: A pore-forming toxin monalysin contributes to infection-induced suppression of defecation in female Drosophila
Source: PLoS Pathog. 2026 Oct 8;22(10):e1014696. doi: 10.1371/journal.ppat.1014696 (PMC13671641; doi:10.1371/journal.ppat.1014696)
Supplement: Supplementary file 3 [file ppat.1014696.s005.docx]

**Manuscript number:** RC-2025-03331

**Corresponding author(s):** Igor Iatsenko

*[Please use this template only if the submitted manuscript should be considered by the affiliate journal as a full revision in response to the points raised by the reviewers.*

*If you wish to submit a preliminary revision with a revision plan, please use our "*[*Revision Plan*](https://docs.google.com/document/u/0/d/1HHp62Tfeb44nd3Gu_3ChB0b8gmMdEog7GjZcZFwHSiQ/edit)*" template.* ***It is important to use the appropriate template to clearly inform the editors of your intentions.****]*

## 1. General Statements [optional]

*This section is optional. Insert here any general statements you wish to make about the goal of the study or about the reviews.*

We thank the reviewers for their favorable evaluation of our work. As you will find in the enclosed point-by-point response, we carefully addressed most of the concerns raised by the reviewers and believe that the revision improved the manuscript. We marked the major text modifications in blue.

## 2. Point-by-point description of the revisions

*This section is mandatory.* *Please insert a point-by-point reply describing the revisions that were already carried out and included in the transferred manuscript.*

Reviewer #1 (Evidence, reproducibility and clarity (Required)):

TITLE
The pore-forming toxin Monalysin contributes to infection-induced suppression of defecation in female Drosophila
________________________________________
SUMMARY
In this study, the authors investigate how Pseudomonas entomophila counteracts increased gut peristalsis, a known antibacterial defense mechanism in Drosophila. They demonstrate that a heat- and protease-sensitive factor mediates reduced defecation following bacterial exposure. Using bacterial mutants, they assess whether major virulence regulators modulate this phenomenon and identify GacA as being involved. A proteomic approach is then employed to identify factors underlying the defecation phenotype, leading the authors to focus on the pore-forming toxin Monalysin. They show that absence of this toxin severely impairs the reduction in defecation following P. entomophila exposure.
While the study addresses an interesting and robust phenotype, the overall approach lacks sufficient rigor and openness toward alternative hypotheses. In its current form, the study is not suitable for publication. Furthermore, the absence of publication of the foundational study (currently available only as a bioRxiv preprint) represents a serious limitation for the present work.
________________________________________
POSITIVE POINTS
• The phenotype described is robust and reproducible.
• The involvement of a heat- and protease-sensitive factor is clearly demonstrated.
• The proteomic strategy and the comparative approach involving hfq and gacA mutants are informative.
________________________________________
NEGATIVE POINTS
• A major weakness lies in the reliance on a pivotal study that remains unpublished. The authors nevertheless use these unpublished data to support the current work.

Response. Our work that identified inhibition of peristalsis by P. entomophila and laid foundations for a present manuscript has been published. Please see the updated reference 22.

• Reduced defecation following P. entomophila exposure may simply reflect reduced food intake. This alternative hypothesis is neither addressed nor tested, either in the current manuscript or in the cited bioRxiv preprint.

R. We quantified food intake (new figures 1E, 4D) and did not find evidence that flies with reduced defecation have reduced intake.

• Although increased peristalsis following pathogen exposure is presented as the entry point of the study, the authors do not directly assess whether peristalsis is indeed reduced under their experimental conditions

R. Direct quantification of intestinal peristalsis (Figure 4E) confirmed that it was reduced after P. entomophila infection.

• The study would benefit from a qualitative analysis of gut morphology.

R. Was performed (Figure 5).

• Overstatement of the conclusions regarding GacA-related data tends to undermine the credibility of the entire study.

R. Corrected.
________________________________________
INTRODUCTION
Minor
Line 66: It is difficult to reconcile the benefit of this phenomenon if it is triggered irrespective of the ingested bacteria. Please clarify this section by detailing how, in adults, this response may benefit the host (e.g., pathogen clearance), while in larvae exposed to L. plantarum it may serve a different purpose.

R. We added a sentence to clarify potential differences between pathogens and microbiota. L70-72.

Major/Minor
Reference 22 is a bioRxiv preprint and has not been peer reviewed. The authors should explicitly state that "a recent preprint suggests...". The data and conclusions from this preprint cannot be used as definitive evidence.

R. Since the paper is published, we left the text unchanged.

Minor
Why was a 2 h incubation at 29{degree sign}C chosen? Please provide a rationale for this relatively high temperature.

R. Experiments were performed following previously-established protocols and 29° C is the temperature close to the optimal growth temperature for P. entomophila.

Major
Reduced defecation may simply reflect reduced feeding. How do the authors control for food intake following bacterial exposure? This should be addressed both qualitatively and quantitatively, at least for the experimental conditions used in Figures 1B and 1C.

R. Food intake was quantified (new figures 1E, 4D).

Major
Putative reductions in peristalsis should be directly tested. Dissected guts from infected adults could be monitored ex vivo in Schneider medium, where peristalsis is easily detectable and quantifiable.

R. Direct quantification of intestinal peristalsis ex vivo was performed as suggested (Figure 4E).

Major
A positive control demonstrating the canonical increase in defecation following exposure to another pathogen (e.g., Ecc15) is necessary, at least in Figure 1B.

R. We used Ecc15 as a positive control (Figure 1B) and indeed observed the expected increase in defecation.

Minor
Line 102: "although still lower than in control flies" - the statistics are non-significant; therefore, no difference should be claimed.

R. The claim was removed.

Minor
Line 103: The experiment is described as testing whether P. entomophila must be alive, yet the results do not discriminate between the need for live bacteria and the requirement for a heat-sensitive product. Only Figure 1D addresses this point. Please rephrase accordingly.

R. Was rephrased.

Minor
Figure 1 legends: Please clearly define the indices used and explain their relevance. For example, how does fold change compare to dots/fly, and how are these values calculated?

R. Explained in lines 103-109. Dots/fly represents the absolute number of defecation spots per fly and therefore shows both treatment effects and variability among experimental days. Fold change represents defecation normalized to the corresponding mean sucrose control from the same experimental day, reducing day-to-day variability and facilitating comparison of relative changes between experimental conditions. Thus, the two representations provide complementary absolute and control-normalized measures of defecation.

Minor
The meaning of "N (days) = ..." is unclear. Please clarify.

R. Clarified in the Figure 1 legend. N (days)” indicates the number of independent experimental days on which the experiment was repeated.

Minor/Major
ANOVA applies to parametric datasets, whereas the Mann-Whitney test does not. No tests are reported to justify the assumption of normality. Please verify whether the datasets are parametric and, if not, apply appropriate non-parametric multiple-comparison tests.

R. We have added the normality test to the Methods section and reanalyzed all results using the appropriate statistical tests according to the distribution of the data.

Major
A qualitative analysis of adult gut morphology would substantially strengthen the study. Transmission light microscopy and actin staining could reveal gut alterations associated with P. entomophila infection and potentially specific effects of Monalysin. This analysis should accompany Figures 1B-1E, 2A-2B, and 4C.

R. We would like to mention that these were technically challenging experiments to perform. Nevertheless, we managed to analyse the most important conditions (Figure 5). We observed that P. entomophila causes gut shrinkage and reduces the intensity of phalloidin staining in a monalysin-dependent manner.
________________________________________
The GacS/GacA system controls secretion of the defecation-blocking factor
Major
Line 121: It is a clear overstatement to claim that "the ΔgacA mutant failed to suppress defecation," as Figure 2B shows a statistically significant ~2-fold decrease compared with sucrose controls. Furthermore, the assertion that "the GacA regulatory system governs secretion or production of" the factor is not supported by the data presented. Such conclusions, which are forced and not supported by the data, cast doubt-and may even discredit-the remainder of the study. Please revise this sentence and consider the entire manuscript with the same level of care.

R. We agree with the reviewer and revised the manuscript accordingly.
________________________________________
Monalysin is one of the factors inhibiting defecation
Minor
Overexpression of the mnl gene in an otherwise innocuous bacterial strain, and assessment of its effect on defecation, would be an informative addition to the study.
R. We expressed monalysin in E. coli using an arabinose-inducible system and observed that such E. coli strain was able to reduce defecation in flies (Figure 4H).

Reviewer #1 (Significance (Required)):

POSITIVE POINTS
• The phenotype described is robust and reproducible.
• The involvement of a heat- and protease-sensitive factor is clearly demonstrated.
• The proteomic strategy and the comparative approach involving hfq and gacA mutants are informative.
________________________________________
NEGATIVE POINTS
• A major weakness lies in the reliance on a pivotal study that remains unpublished. The authors nevertheless use these unpublished data to support the current work.
• Reduced defecation following P. entomophila exposure may simply reflect reduced food intake. This alternative hypothesis is neither addressed nor tested, either in the current manuscript or in the cited bioRxiv preprint.
• Although increased peristalsis following pathogen exposure is presented as the entry point of the study, the authors do not directly assess whether peristalsis is indeed reduced under their experimental conditions.
• The study would benefit from a qualitative analysis of gut morphology.
• Overstatement of the conclusions regarding GacA-related data tends to undermine the credibility of the entire study.

If fully corrected, the study will interest specialists in host-pathogens interactions with invertebrates as hosts.


Reviewer #2 (Evidence, reproducibility and clarity (Required)):

The manuscript by Rubinić et al. investigates how the Drosophila pathogen Pseudomonas entomophila (Pe) suppresses host defecation, a defense mechanism that normally promotes pathogen clearance. Building on prior observations that Pe induces a sex-specific blockade of defecation, the authors identify a secreted, thermosensitive protein factor regulated by the GacS/GacA two-component system. Through comparative proteomics and functional assays, they implicate the pore-forming toxin Monalysin as a contributor to infection-induced suppression of defecation.
The experimental approach is well designed, combining genetic and proteomic analyses with a quantitative physiological readout. The data are convincing, clearly presented, and support the main conclusions. The proteomic analysis is thorough; however, the rationale for prioritizing Monalysin among the 146 candidate proteins could be made more explicit. While Monalysin is a logical choice given prior knowledge of its role in virulence and epithelial damage, briefly discussing why other prominent candidates were not pursued would improve transparency and guide future work.

R. We thank the reviewer for constructive feedback. We provide additional justification for our selection of Monalysin. L190-193.

The authors show that deletion of mnl significantly attenuates defecation suppression but does not fully abolish it, indicating that Monalysin is only a partial contributor to this phenotype. This point is appropriately acknowledged in the manuscript, but the interpretation would benefit from expanding on the discussion of additional candidate proteins highlighted in Figure 4. Ideally other candidates from that list should be tested.

R. We extended the discussion on additional factors. L278-287.

Although the identification of Monalysin is convincing, the mechanistic link between pore formation and reduced gut motility remains unclear. The study would benefit from further characterization of how Monalysin affects gut homeostasis, including potential connections to known pathways such as TRPA1 signaling.

R. We agree that further characterization of how Monalysin inhibits gut peristalsis would be valuable. Given the numerous possible mechanisms, we consider a detailed investigation of this aspect to be beyond the scope of the present study and therefore leave it for future studies. We have expanded the Discussion to address potential mechanisms by which Monalysin may affect gut motility. L267-274.

Moreover, linking defecation impairment more directly to virulence outcomes, for instance by including survival curves comparing infections with wild-type and Δmnl bacteria, would help clarify the physiological relevance of this phenotype.

R. We included the survival curve and also pathogen load (Fig 4F, 4G).

Several points of clarification would further improve reproducibility and clarity. The authors should specify the CFU amounts used in infection experiments and clarify how these relate to the preparation of bacterial supernatants. It would also be helpful to indicate whether the effect of the supernatant on defecation is dose dependent. In addition, while the fold-change panels included in several figures are informative, they are somewhat redundant, and briefly justifying their inclusion in the figure legends or explaining how they complement the absolute defecation counts would improve clarity.
R. We have added the CFU counts in the methods. The relevance of fold change panels has been explained (see response to reviewer 1).

Reviewer #2 (Significance (Required)):

This work extends the known functions of Monalysin and provides a conceptual advance in understanding how pathogens can subvert host defenses by modulating intestinal transit rather than simply triggering inflammatory or cytotoxic responses. Impairment of defecation represents a broadly used pathogenic strategy. In this context, the manuscript would benefit from a broader comparative perspective, such as a genomic and/or genetic analysis of mnl to assess its conservation and variability across P. entomophila strains or related pathogens. Such an analysis would help place the findings within a wider evolutionary framework and strengthen the argument that suppression of gut motility is a common and adaptive microbial strategy.
Overall, this study should be of broad interest to researchers working on host-microbe interactions, gut physiology, and bacterial pathogenesis, and it provides a strong foundation for future mechanistic and comparative work in this area.
R. We thank the reviewer for the suggestions. To provide a broader comparative perspective, we performed a phylogenetic analysis of Monalysin-related proteins from multiple *P. entomophila* strains and related bacterial isolates. The analysis shows that Monalysin from *P. entomophila* strains cluster closely, whereas related proteins are also present in other *Pseudomonas* isolates and more distantly related bacteria. We have incorporated these findings into the revised manuscript (Fig. 6; lines 244–255).

Reviewer #3 (Evidence, reproducibility and clarity (Required)):

Summary:

The authors were interested in the mechanisms by which the entomopathogenic Pseudomonas entomophila bacterium can inhibit defecation in Drosophila. Using a differential proteomic approach, they identified Monalysin, a secreted pore-forming toxin, as a good candidate. They further showed thanks to the use of a P. entomophila mutant deleted for the mnl gene, that Mnl was indeed involved in suppressing host defecation.


Major comments:

Although overall the experiments were well designed and analyzed, one key experiment is lacking to fully support their conclusion:
The authors claim (for instance lines 195-196) that "Monalysin as one of the bacterial factors required for suppressing defecation and bacterial clearance".
The author have to demonstrate the bacterial clearance. They can perform CFU counting (bacterial load monitoring) in the gut infected with WT Pe vs Delta-mnl mutant. This experiment is essential to state that the blockage of defecation slow down bacterial clearance. Such data would strengthened their study.

R. CFU counts are included and showed reduced persistence (Fig 4F).

Minor comments:

- Line 102: "...although still lower than in control flies (Fig. 1B, Fig. 1B')." Authors should add "although not significant".
R. The sentence was rewritten.

- It would be very useful for readers and future experimenters to know the amount (in CFU) of P. entomophila provided per fly. The authors indicate a bacterial concentration in OD600 (lines 273-275). However, an OD does not reflect the quantity/concentration of bacteria. For a given volume at a given OD600 value, each bacterium has a different concentration (mainly due to the size of the bacteria). Also, the number of flies varies from 10 to 20 per vial. Does the amount of bacteria provided vary according to the number of flies?

R. We quantified colony-forming units (CFUs) of Pseudomonas entomophila and report these values in the Methods. We used the same infection mixture—and thus the same bacterial concentration—for vials containing either 10 or 20 flies. Given the high bacterial concentration, flies in both conditions ingested an equal number of bacteria, as shown in the graph below.

*Amount of ingested P. entomophila cells depending on the number of flies in the vial. 10 or 20* *flies per vial were infected with P. entomophila OD200 mixed 1:1 with sucrose for 0.5 h. CFUs were quantified in single flies by plating the serially diluted homogenate on LB plates. Data are shown as log10 CFU/fly.*

- Lines 119-123 and figures 2B, B': how do the authors explain that in Delta-gacA Pe mutant, the defecation is only partially restored? The authors should mitigate their conclusion lines 122-123 as well as in the rest of the manuscript.

R. We agree and have revised the manuscript accordingly.

Moreover, they stated lines 182-186 that Delta-mnl mutant only partially suppress defecation. If we compare figure 4C-C' to figure 2 B-B', it seems that delta-mnl has a stronger phenotype than Delta-gacA. In other word, Mnl is likely only partially regulated by GacA since removing Mnl has a stronger effect on defecation rescue than removing GacA.

R. We agree and mentioned this.

- Lines 492-493: Incomplete reference.
R. Corrected

Reviewer #3 (Significance (Required)):

Defecation has been previously described as participating to the elimination of pathogens. Understanding how pathogens hijack host defenses is of prime importance to combat them. In this study, the authors identified a secreted factor, Monalysin, implicated in the inhibition of the defecation in Drosophila. Monalysin has been previously characterized as a pore-forming toxin damaging the gut of Drosophila. The data presented here, unravel a "side" function of this toxin. Although Monalysin is probably not the only factor of P. entomophila involved in blocking defecation, the data presented provide information on virulence mechanisms and the versatility of a toxin and could help the "Host-Pathogen" community to better understand microbial virulence.

My expertise: host-pathogen interaction using Drosophila as host model.
