# Supplementary material for: A pore-forming toxin monalysin contributes to infection-induced suppression of defecation in female Drosophila
Source: PLoS Pathog. 2026 Oct 8;22(10):e1014696. doi: 10.1371/journal.ppat.1014696 (PMC13671641; doi:10.1371/journal.ppat.1014696)
Supplement: Peer review history

# Author comment: Author response to Decision Letter 0

**DOI:** 10.1371/journal.ppat.1014696.r001

26 Aug 2026

Attachment Submitted filename: 164214_1_rebuttal_3850866_tjybp3.docx

# Peer review: Decision Letter 0

**DOI:** 10.1371/journal.ppat.1014696.r002

**Authors:** David Skurnik

18 Sep 2026

PPATHOGENS-D-26-02015

A Pore-Forming Toxin Monalysin Contributes to Infection-Induced Suppression of Defecation in Female Drosophila

PLOS Pathogens

Dear Dr. Iatsenko,

Thank you for submitting your manuscript to PLOS Pathogens. After careful consideration, we feel that it has merit but does not fully meet PLOS Pathogens's publication criteria as it currently stands. Therefore, we invite you to submit a revised version of the manuscript that addresses the points raised during the review process.

Please submit your revised manuscript by Oct 18 2026 11:59PM. If you will need more time than this to complete your revisions, please reply to this message or contact the journal office at plospathogens@plos.org. When you're ready to submit your revision, log on to https://www.editorialmanager.com/ppathogens/ and select the 'Submissions Needing Revision' folder to locate your manuscript file.

Please include the following items when submitting your revised manuscript:

* A letter that responds to each point raised by the editor and reviewer(s). You should upload this letter as a separate file labeled 'Response to Reviewers'. This file does not need to include responses to any formatting updates and technical items listed in the 'Journal Requirements' section below.

* A marked-up copy of your manuscript that highlights changes made to the original version. You should upload this as a separate file labeled 'Revised Manuscript with Track Changes'.

* An unmarked version of your revised paper without tracked changes. You should upload this as a separate file labeled 'Manuscript'.

If you would like to make changes to your financial disclosure, competing interests statement, or data availability statement, please make these updates within the submission form at the time of resubmission. Guidelines for resubmitting your figure files are available below the reviewer comments at the end of this letter.

As the corresponding author, your ORCID iD is verified in the submission system and will appear in the published article. PLOS supports the use of ORCID, and we encourage all coauthors to register for an ORCID iD and use it as well. Please encourage your coauthors to verify their ORCID iD within the submission system before final acceptance, as unverified ORCID iDs will not appear in the published article. Only the individual author can complete the verification step; PLOS staff cannot verify ORCID iDs on behalf of authors.

We look forward to receiving your revised manuscript.

Kind regards,

David Skurnik, M.D., Ph.D.

Section Editor

PLOS Pathogens

Sumita Bhaduri-McIntosh

Editor-in-Chief

PLOS Pathogens

orcid.org/0000-0003-2946-9497

Dominique Soldati-Favre

Editor-in-Chief

PLOS Pathogens

orcid.org/0000-0003-4156-2109

**Additional Editor Comments (if provided):**

**Journal Requirements:**

1. We noted that you have included an author contributions statement in your manuscript file, and it appears to differ from the information we have on file in your submission form. Please remove the statement from your manuscript and instead ensure that the full contributions of each author are acknowledged in the "Add/EditRemove Authors" section of our submission form.

The list of CRediT author contributions may be found here: https://journals.plos.org/plospathogens/s/authorship#loc-author-contributions

2. Please provide a detailed Financial Disclosure statement. This is published with the article. It must therefore be completed in full sentences and contain the exact wording you wish to be published.

- State what role the funders took in the study. If the funders had no role in your study, please state: “The funders had no role in study design, data collection and analysis, decision to publish, or preparation of the manuscript.”

- If any authors received a salary from any of your funders, please state which authors and which funders.

3. Your current Financial Disclosure states, “Deutsche Forschungsgemeinschaft (DFG):Igor Iatsenko IA 81/2-1; Deutsche Forschungsgemeinschaft (DFG):Igor Iatsenko IA81/3-1; Boehringer Ingelheim Stiftung (Boehringer Ingelheim Foundation):Igor Iatsenko”. However, your funding information on the submission form indicates that you received funding from “Max-Planck-Gesellschaft  (Dr Igor Iatsenko)”. Please indicate by return email the full and correct funding information for your study and confirm the order in which funding contributions should appear. Please be sure to indicate whether the funders played any role in the study design, data collection and analysis, decision to publish, or preparation of the manuscript.

4. Please provide separate figure files in .tif or .eps format.

For more information about figure files please see our guidelines: https://journals.plos.org/plospathogens/s/figures

https://journals.plos.org/plospathogens/s/figures#loc-file-requirements

5. Please provide an Author Summary. This should appear in your manuscript between the Abstract (if applicable) and the Introduction, and should be 150–200 words long. The aim should be to make your findings accessible to a wide audience that includes both scientists and non-scientists. Sample summaries can be found on our website under Submission Guidelines: https://journals.plos.org/plospathogens/s/submission-guidelines#loc-parts-of-a-submission

6. We do not publish any copyright or trademark symbols that usually accompany proprietary names, eg (R), (C), or TM  (e.g. next to drug or reagent names). Please remove all instances of trademark/copyright symbols throughout the text, including Fluor® on page 18 and Pierce™ on page 20.

7. We notice that your supplementary figures are included in the manuscript file. Please remove them and upload them with the file type 'Supporting Information'. Please ensure that each Supporting Information file has a legend listed in the manuscript after the references list.

8. Some material included in your submission may be copyrighted. According to PLOS’s copyright policy, authors who use figures or other material (e.g., graphics, clipart, maps) from another author or copyright holder must demonstrate or obtain permission to publish this material under the Creative Commons Attribution 4.0 International (CC BY 4.0) License used by PLOS journals. Please closely review the details of PLOS’s copyright requirements here: PLOS Licenses and Copyright. If you need to request permissions from a copyright holder, you may use PLOS's Copyright Content Permission form.

Please respond directly to this email and provide any known details concerning your material's license terms and permissions required for reuse, even if you have not yet obtained copyright permissions or are unsure of your material's copyright compatibility. Once you have responded and addressed all other outstanding technical requirements, you may resubmit your manuscript within Editorial Manager.

**Potential Copyright Issues:**

Figure 4A: Please confirm whether you drew the images / clip-art within the figure panels by hand. If you did not draw the images, please provide (a) a link to the source of the images or icons and their license / terms of use; or (b) written permission from the copyright holder to publish the images or icons under our CC BY 4.0 license. Alternatively, you may replace the images with open source alternatives. See these open source resources you may use to replace images / clip-art:

- https://commons.wikimedia.org

- https://openclipart.org/

If the reviewer comments include a recommendation to cite specific previously published works, please review and evaluate these publications to determine whether they are relevant and should be cited. There is no requirement to cite these works unless the editor has indicated otherwise.

[Note: HTML markup is below. Please do not edit.]

**Reviewers' Comments:**

Reviewer's Responses to Questions

**Part I - Summary**

Please use this section to discuss strengths/weaknesses of study, novelty/significance, general execution and scholarship.

Reviewer #1: From the submission in Review Commons, all my comments were answered, tested and discussed, thanks a lot.

The manuscript is really improved this way.

Reviewer #2: Defecation is a mechanism that contributes to an organism's defense against ingested pathogens. Accelerated defecation favors the elimination pathogens, whereas slowed defecation promotes their persistence.

The authors identified the Monalysin toxin from Pseudomonas entomophila as a key factor in blocking defecation. Their experiments clearly demonstrate the role of this toxin in inhibiting muscle contractions and the persistence of bacteria, which is associated with higher mortality.

The authors have fully addressed the questions I had raised. They also included the requested experiments. Considering the additional experiments, the supplementary information provided, and the revisions made to address the queries from the other two reviewers, I believe this work deserves to be published in PLOS Pathogens.

Reviewer #3: I thank the authors for addressing my comments and for the additional analyses included in the revised manuscript. In particular, the authors have clarified the rationale for selecting Monalysin from the proteomic candidates, expanded the discussion of other potential factors, added data addressing the physiological relevance of the phenotype, clarified the infection conditions and CFU amounts, and provided a comparative phylogenetic analysis of Monalysin homologues.

The revisions adequately address my concerns, and the manuscript has been substantially improved. I am satisfied with the authors’ responses and have no further major comments.

I therefore support publication of the manuscript in its revised form.

**********

**Part II – Major Issues: Key Experiments Required for Acceptance**

Please use this section to detail the key new experiments or modifications of existing experiments that should be absolutely required to validate study conclusions.

Generally, there should be no more than 3 such required experiments or major modifications for a "Major Revision" recommendation. If more than 3 experiments are necessary to validate the study conclusions, then you are encouraged to recommend "Reject".

Reviewer #1: (No Response)

Reviewer #2: None. The authors have addressed all of my previous questions and suggestions.

Reviewer #3: (No Response)

**********

**Part III – Minor Issues: Editorial and Data Presentation Modifications**

Please use this section for editorial suggestions as well as relatively minor modifications of existing data that would enhance clarity.

Reviewer #1: *P.e. and F-actin in the guts:

RT-qPCR or Western blot quantif of F-actin in guts from sucrose fed vs guts from P.e. fed animals.

>Will not change the conclusions, but may pave the way to study this extremely strong phenotype.

*1E and 4D, food intake legend: please indicate that the set of data is the same in both figures.

*Please mention directly in the text why 29°c was choosen while it is a stressful temparature for the flies.

Reviewer #2: None. The authors have addressed all of my previous questions and suggestions.

Reviewer #3: (No Response)

**********

PLOS authors have the option to publish the peer review history of their article (what does this mean?). If published, this will include your full peer review and any attached files.

If you choose “no”, your identity will remain anonymous but your review may still be made public.

**Do you want your identity to be public for this peer review?**  For information about this choice, including consent withdrawal, please see our Privacy Policy.

Reviewer #1: No

Reviewer #2: **Yes:**  Armel GALLET

Reviewer #3: No

[NOTE: If reviewer comments were submitted as an attachment file, they will be attached to this email and accessible via the submission site. Please log into your account, locate the manuscript record, and check for the action link "View Attachments". If this link does not appear, there are no attachment files.]

**Figure resubmission:**

**Reproducibility:**

To enhance the reproducibility of your results, we recommend that authors of applicable studies deposit laboratory protocols in protocols.io, where a protocol can be assigned its own identifier (DOI) such that it can be cited independently in the future. Additionally, PLOS ONE offers an option to publish peer-reviewed clinical study protocols. Read more information on sharing protocols at https://plos.org/protocols?utm_medium=editorial-email&utm_source=authorletters&utm_campaign=protocols

# Author comment: Author response to Decision Letter 1

**DOI:** 10.1371/journal.ppat.1014696.r003

25 Sep 2026

Attachment Submitted filename: Point by point response.docx

# Editor report: Decision Letter 1

**DOI:** 10.1371/journal.ppat.1014696.r004

**Authors:** David Skurnik

3 Oct 2026

Dear Dr Iatsenko,

We are pleased to inform that your manuscript, "A Pore-Forming Toxin Monalysin Contributes to Infection-Induced Suppression of Defecation in Female Drosophila", has been editorially accepted for publication at PLOS Pathogens.

Before your manuscript can be formally accepted and sent to production, you will need to complete our formatting changes, which you will receive by email within a week. Please note that your manuscript will not be scheduled for publication until you have made the required changes.

IMPORTANT NOTES

(1) Copyediting and Proofreading: The corresponding author will receive a typeset proof for review, to ensure errors have not been introduced during production. Please review the PDF proof of your manuscript carefully, as this is the last chance to correct any errors. Please note that major changes, or those which affect the scientific understanding of the work, will likely cause delays to the publication date of your manuscript.

(2) Appropriate Figure Files: Please remove all name and figure # text from your figure files. Please also take this time to check that your figures are of high resolution, which will improve the readbility of your figures and help expedite your manuscript's publication. Please note that figures must have been originally created at 300dpi or higher. Do not manually increase the resolution of your files. For instructions on how to properly obtain high quality images, please review our Figure Guidelines, with examples at: http://journals.plos.org/plospathogens/s/figures.

We encourage you to use PACE (http://pace.apexcovantage.com/), a figure-checking tool which helps authors ensure all figures meet PLOS requirements so that figures can publish in the highest quality. PACE is a digital diagnostic and conversion tool for figure files. It will provide information about any failed check(s) and, if able, will automatically convert the figure into an acceptable file that passes quality checks.

To use PACE, you must first register as a user. Then, login and navigate to the UPLOAD tab, where you will find detailed instructions on how to use the tool.

(3) Striking Image: Please upload a striking still image to accompany your article if one is available (you can include a new image or an existing one from within your manuscript). Should your paper be accepted, this image will be considered for our monthly issue image and may also appear on our website to feature your article. Please upload this as a separate file, selecting "striking image" as the file type upon upload. Please also include a separate "Other" file with a caption, including credits and any potential copyright information. Please do not include the caption in the main article file. If your image is from someone other than yourself, please ensure that the artist has read and agreed to the terms and conditions of the Creative Commons Attribution License at http://journals.plos.org/plospathogens/s/content-license. Please note that PLOS cannot publish copyrighted images.

(4) Press Release or Related Media: If you or your institution will be preparing press materials for this manuscript, please inform our press team in advance at plospathogens@plos.org as soon as possible. We ask that you contact us within one week to plan ahead of our fast Production schedule. If you need to know your paper's publication date for related media purposes, you must coordinate with our press team, and your manuscript will remain under a strict press embargo until the publication date and time.

(5) Update your Profile Information: Now that your manuscript has been provisionally accepted, please log into Editorial Manager and update your profile, if needed. Go to http://www.editorialmanager.com/ppathogens, log in, and click on the "Update My Information" link at the top of the page. Please update your user information to ensure an efficient production and billing process.

(6) LaTeX users only: Our staff will ask you to upload a TEX file in addition to the PDF before the paper can be sent to typesetting, so please carefully review our Latex Guidelines http://journals.plos.org/plospathogens/s/latex in the meantime.

Thank you again for supporting Open Access publishing; we are looking forward to publishing your work in PLOS Pathogens.

Best regards,

David Skurnik, M.D., Ph.D.

Section Editor

PLOS Pathogens

David Skurnik

Section Editor

PLOS Pathogens

Dominique Soldati-Favre

Editor-in-Chief

PLOS Pathogens

***********************************************************

# Editor report: Acceptance letter

**DOI:** 10.1371/journal.ppat.1014696.r005

**Authors:** David Skurnik

Dear Dr Iatsenko,

We are delighted to inform you that your manuscript, "A Pore-Forming Toxin Monalysin Contributes to Infection-Induced Suppression of Defecation in Female Drosophila," has been formally accepted for publication in PLOS Pathogens.

We have now passed your article onto the PLOS Production Department who will complete the rest of the pre-publication process. All authors will receive a confirmation email upon publication.

Thank you again for supporting open-access publishing; we are looking forward to publishing your work in PLOS Pathogens.

Best regards,

Dominique Soldati-Favre

Editor-in-Chief

PLOS Pathogens
